# Estimated atrial fibrillation burden on early rhythm-control and cardiovascular events in the EAST-AFNET 4 trial

**DOI:** 10.1016/j.eclinm.2025.103457

**Published:** 2025-09-01

**Authors:** Stef Zeemering, Katrin Borof, Ulrich Schotten, Julius Obergassel, A John Camm, Harry J.G.M. Crijns, Lars Eckardt, Larissa Fabritz, Andreas Goette, Zarina Habibi, Jordi Heijman, Ben J.M. Hermans, Marc D. Lemoine, Christina Magnussen, Andreas Metzner, Andreas Rillig, Renate B. Schnabel, Eva Schuijt, Anna Suling, Panos Vardas, Stephan Willems, Antonia Zapf, Paulus Kirchhof

**Affiliations:** a Department of Physiology, Cardiovascular Research Institute Maastricht (CARIM), Maastricht University, Maastricht, the Netherlands; b Department of Cardiology, University Medical Center Hamburg-Eppendorf, Hamburg, Germany; c Department of Cardiology, Cardiovascular Research Institute Maastricht (CARIM), Maastricht University Medical Center, Maastricht, the Netherlands; d Atrial Fibrillation NETwork (AFNET), Mendelstr. 11, Münster 48149, Germany; e University Center of Cardiovascular Science (UCCS), University Medical Center Hamburg Eppendorf, Hamburg, Germany; f German Centre for Cardiovascular Research (DZHK), Partner Site Hamburg/Kiel/Luebeck, Germany; g Clinical Sciences, City St George’s University, London, UK; h Department of Cardiology II (Electrophysiology), University Hospital Münster, Germany; i Institute of Cardiovascular Sciences, University of Birmingham, Birmingham, UK; j Department of Cardiology and Intensive Care Medicine, St. Vincenz Hospital, Paderborn, Germany; k Medical Faculty, Otto-von-Guericke University, Magdeburg, Germany; l Division of Medical Physics and Biophysics, Gottfried Schatz Research Center, Medical University of Graz, Graz, Austria; m Center for Population Health Innovation (POINT), University Medical Center Hamburg-Eppendorf, Hamburg, Germany; n Department of Cardiology, Heraklion University Hospital, Heraklion, Crete, Greece; o Biomedical Research Foundation Academy of Athens (BRFAA), Greece and Hygeia Hospitals Group, Athens, Greece; p Department of Cardiology, Hospital St Georg, Hamburg, Germany; q Institute of Medical Biometry and Epidemiology, University Medical Center Hamburg-Eppendorf, Hamburg, Germany

**Keywords:** Atrial fibrillation, Patient-operated ECG, Atrial fibrillation burden, Rhythm-control therapy, Stroke, Heart failure, Cardiovascular death

## Abstract

**Background:**

Atrial fibrillation (AF) is currently diagnosed by ECG, creating a binary, lifelong diagnosis. AF burden, estimated as the proportion of time spent in AF, quantifies AF severity dynamically. AF burden can modulate the risk of AF-related outcomes. Whether AF burden modulates cardiovascular outcomes with rhythm-control therapy is unknown.

**Methods:**

AF burden on early rhythm-control was estimated using supervised artificial-intelligence-based rhythm classification of patient-operated telemetric short-term ECGs in patients randomised to early rhythm-control in the EAST-AFNET 4 trial (NCT01288352, ISRCTN04708680, conducted between 2011 and 2020). ECGs were transmitted 1–2 times per week and during symptoms. A landmark was set at 12 months and efficacy and safety outcomes occurring during the subsequent 4.1 years of follow-up were compared by estimated AF burden quartiles (Q1–Q4).

**Findings:**

In 1178 patients (70 years, 47% women, CHA_2_DS_2_-VA 2.8 ± 1.2) transmitting 303,308 ECGs over 5.1 years, (median 1/week, IQR 1; 2) estimated AF burden was 6% [0%; 22%] in the first year of follow-up. Estimated AF burden below the median was associated with low rates of cardiovascular death, stroke, or unplanned hospitalisation for heart failure or acute coronary syndrome (Q1: 2.0 events/100 patient-years; Q2: 2.6 events/100 patient-years). A higher estimated AF burden was associated with higher event rates (Q3: 4.8 events/100 patient-years; Q4: 4.2 events/100 patient-years), comparable to events with usual care (4.5 events/100 patient-years). Sensitivity analyses confirmed these findings.

**Interpretation:**

These hypothesis-generating findings suggest that AF burden estimated by weekly short-term patient-operated ECGs modulates AF-related events on rhythm-control therapy. Pending validation and evaluation of residual confounding, estimation of AF burden can refine AF diagnosis.

**Funding:**

EAST-AFNET4 was supported by a grant from the German Ministry of Education and Research (01 GI0204) via the German Center for Cardiovascular Research (DZHK), the 10.13039/100020028Atrial Fibrillation NETwork (AFNET), the European Heart Rhythm Association, 10.13039/100006279St. Jude Medical/Abbott, Sanofi, and the German Heart Foundation. These analyses received additional support from the 10.13039/501100000780European Union (grant agreement 965286 [MAESTRIA]), British Heart Foundation (AA/18/2/34218), German Center for Cardiovascular Research supported by the German Ministry of Education and Research (DZHK, grant numbers DZHK FKZ 81X2800182, 81Z0710116, and 81Z0710110), 10.13039/501100001659German Research Foundation (Ki 509167694) and the 10.13039/501100003042Else Kröner-Fresenius Foundation, Dutch Heart Foundation (Grant number 01-002-2022-0118, EmbRACE), and the Leducq Foundation (2024, Immune Targets for Atrial Fibrillation).

**Research in context**

**Evidence before this study**

We searched PubMed for English-language articles using the search term “atrial fibrillation burden” AND “stroke” OR “heart failure” OR “death” OR “outcomes”, from database inception to May 22nd, 2025. While the diagnosis of AF is currently made in a binary fashion based on a single ECG, newer data suggest that AF burden, defined as the time spent in AF per monitored time, is associated with stroke and other cardiovascular events, supporting its role as a quantitative parameter to guide and individualise management of patients with AF. Whether AF burden reduction is associated with fewer cardiovascular events is not known.

**Added value of this study**

This hypothesis-generating landmark analysis within the early rhythm-control group of the EAST-AFNET 4 trial suggests that the outcome-reducing effect of early rhythm control therapy is modulated by estimated AF burden calculated from weekly short-term patient-operated ECGs over a year: A low estimated burden in the first year of therapy was associated with 50% fewer cardiovascular events in the subsequent 4 years of follow-up. A high AF burden on therapy was associated with higher event rates that were comparable to event rates in the comparator arm, symptom-directed rhythm control.

**Implications of all the available evidence**

These pilot findings suggest that AF burden estimated by weekly patient-operated ECGs over a year can assess the effects of early rhythm control therapy on mid-term cardiovascular events. The findings also suggest that patient-operated rhythm monitors can, in certain conditions, be used to estimate AF burden. Pending external validation and evaluation of residual confounding, the findings pave the way for integration of AF burden into digital tools to refine AF diagnosis and to guide personalised AF therapy.

## Introduction

Atrial fibrillation (AF) is a common heart rhythm disorder that has a high potential for improved management employing digital medicine.^1^ Successful adoption of digital tools and artificial intelligence (AI) into care requires clearly defined, measurable outcomes or surrogate markers to quantify efficacy, safety, and to enable broad, efficient access to technology. Recent data suggest that the proportion of time spent in AF, called AF burden, influences the risk of AF-related complications such as stroke,^2^ heart failure hospitalizations, cardiovascular death and others. AF detected by ECG screening^3^ or by screening using implanted loop recorders,^4^ as well as device-detected AF^5^^,^^6^ are all associated with a low AF burden and a low risk of stroke. The outcome-reducing effects of AF ablation are associated with a reduced AF burden in patients with AF and severe heart failure.^7^ These data suggest that knowledge about the AF burden can enhance the diagnosis of AF using quantitative, objective information.^8^ The current ACC/AHA/HRS guideline committee proposed AF burden reduction as a potential therapeutic goal in patients with AF but highlighted evidence gaps blocking adoption of this concept into clinical care.^9^

The most precise method to quantify AF burden is 24/7 monitoring using implantable loop recorders or cardiac implanted electronic devices.^4^^,^^7^^,^^10^ Intermittent monitoring using patient-operated ECGs or consumer-electronic rhythm monitors provides less precise estimates of AF burden with a similar directionality as AF burden quantified by continuous rhythm monitoring.^3^^,^^8^ Frequent daily short-term ECG recording have a similar precision for AF detection to repeated 24-h Holter ECG recordings,^11^ suggesting that regular short-term ECG recordings could be used to estimate AF burden. If AF burden estimates by patient-operated ECGs were related to cardiovascular outcomes, this would invite their use for remote, digital patient management.^8^

Early-rhythm-control therapy, initiated within one year after a first diagnosis of AF, reduced the risk of cardiovascular events by 21% in the *Early Treatment of Atrial Fibrillation for Stroke Prevention Trial* (EAST-AFNET 4), including fewer strokes and heart failure events.^12^ To determine the value of AF burden estimated by patient-operated ECGs for these outcomes, this study applied a digital algorithm to determine rhythm in patient-operated electrocardiograms (ECGs) transmitted from patients randomised to early rhythm-control in the EAST-AFNET 4 trial. In the trial, all patients assigned to early rhythm-control were asked to transmit a patient-operated ECG twice per week and whenever symptoms occurred. In this analysis, this information was used to estimate AF burden. Estimated AF burden on early rhythm-control was related to the main trial outcomes.

## Methods

### Study design

This is a prespecified analysis of the EAST-AFNET 4 data set and the first to report analysis of its telemetric ECGs. EAST-AFNET 4, an investigator-initiated, parallel-group, open, blinded-outcome-assessment trial, randomised 2789 adults aged over 75 years, or who had a previous stroke or transient ischemic attack or a defined comorbidity profile, within 12 months after first AF diagnosis to early rhythm-control or usual care.^12^ Patients randomised to early rhythm-control were asked to transmit two ECGs per week and whenever they felt symptoms.^12^ Telemetric ECGs were recorded using a Vitaphone 100 device (Vitaphone, Munich, Germany) and transmitted to a central data server for further analysis via telephone lines.^12^ ECG monitoring triggered automated alerts for centers upon transmission of abnormal ECGs. EAST-AFNET 4 was planned and conducted by the Atrial Fibrillation Network (AFNET). The protocol was approved by the ethics review boards at all institutions involved. Written informed consent was provided by all patients who participated in the trial. This analysis estimates AF burden in telemetric ECGs in patients randomised to early rhythm control and relates it to cardiovascular outcomes.

### Randomization and masking

Patients were randomly assigned to receive early rhythm control or usual care using an electronic case report form. Treatment allocation was open-label. All clinical events were adjudicated by an independent, blinded endpoint review committee. Statistical analyses were performed by blinded statisticians not involved in patient care. Telemetric ECG monitoring was part of early rhythm-control.

### Ethics

The EAST-AFNET 4 trial was approved by ethics committees in all participating sites and countries. All patients gave written informed consent prior to participation in the trial. This analysis was prespecified in the analysis plan and includes post-hoc elements.

### Artificial intelligence-enabled AF burden estimation from patient-operated ECGs

Supervised machine learning and deep learning classifiers were trained for noise detection and rhythm classification in telemetric ECGs utilizing 3587 high-quality annotations. ECGs in the noise-cleaned dataset were categorised as AF or atrial flutter, sinus rhythm (SR), or other rhythm, with the latter being excluded from analysis. We provide an artificial intelligence checklist in the Supplementary information as recommended by the European Heart Rhythm Association (EHRA).^13^

### Data processing and annotation

Electrocardiogram (ECG) data consisted of patient-operated, single lead, 30-s ECGs (VitaPhone). In total 304,089 ECGs were recorded by 1236 patients who received early rhythm-control in the EAST-AFNET 4 trial. ECG signals and recording date were automatically extracted from the ECG stored in a portable document format. ECGs were resampled to a sampling frequency of 200 Hz. Two subsets of the ECGs were sampled for manual rhythm annotation and rhythm classifier training and testing. The training set was enriched for ECGs with fast and slow heart rates with irregular ECG patterns. To determine heart rate, QRS timing was detected in each ECG using the *jqrs* method.^14^ ECG irregularity was determined by recurrence plot analysis.^15^ Recurrence plots were constructed by computing the similarity (one minus the cosine distance) between all 0.5 s ECG segments. Segments pairs with a maximum similarity value of 0.15 were considered a re-occurrence of the same short-term ECG pattern. ECG irregularity was defined as one minus the relative number of recurrences, a number between 0 and 1. The joint distribution of these two measures (heart rate and irregularity) over all ECGs was discretised into 10 × 10 equally spaced bins. Only bins containing at least 500 observations were considered in the sampling procedure, with equal probability. For each patient, two ECGs, if available, were sampled from two random bins. If a patient did not have any ECGs in eligible bins, ECGs were sampled randomly. The test set was created by randomly sampling a third ECG for each patient, when available. Rhythm was annotated by 4 independent observers (SZ, BH, ZH, and US) by visual inspection of the sampled ECGs, supported by a tachogram and Poincaré plot of the RR-interval series. Rhythm was labeled as normal sinus rhythm, atrial fibrillation or flutter (AF) or other. Other rhythms included non-AF irregular rhythms, for instance due to multiple premature ventricular or atrial complexes. ECGs in which the rhythm could not be accurately assessed visually due to poor signal quality were labeled as Noise. Examplary ECGs are shown in Supplementary Fig. S1. The distribution of different rhythms is shown in Supplementary Table S1.

### Rhythm classification model design and implementation

Rhythm classification was implemented as a two-stage procedure, in which first noisy ECGs were detected and excluded, and then a three-class classifier performed the rhythm classification. A convolutional neural network (CNN) was implemented to detect noisy ECGs, consisting of 3 blocks of 1-D convolution (64 filters with size 16), rectified linear unit, batch normalization and max pooling (stride 2) layers, followed by two fully connected layers (output size 64 and 32) and a SoftMax layer. CNN input were ECGs filtered with a 0.5–40 Hz bandpass filter and standardised to zero mean and unit variance. The CNN was trained by optimizing the binary-cross entropy using the Adam solver. A support vector machine (SVM) classifier was implemented to classify the three rhythm categories (SR = sinus rhythm, AF = atrial fibrillation, and O = other rhythm) based on 13 commonly used RR-interval features for AF detection^16^:

- RR-interval mean, median, standard deviation, maximum, and minimum
- Percentage of RR intervals larger than 50 ms and the square root of the mean squared difference of consecutive RR intervals
- RR interval coefficient of sample entropy^17^ and fuzzy measure entropy^18^
- Median absolute deviation from the mean heart rate of three adjacent RR intervals of similar length
- Normalised low frequency (0.04–0.15 Hz) and high frequency (0.15–0.40 Hz) power of the RR interval series, and their ratio.

The SVM kernel was optimised for maximum performance using rhythm-stratified 5-fold cross validation. All analyses were performed in MATLAB R2024b, Natick, Massachusetts: The MathWorks Inc. 2024. The performance of both classifiers in the two-staged procedure was assessed on the annotated training and test sets. Performance was quantified as accuracy, area under the receiver operating characteristics curve (AUC) and the F1-score.

### Definition of AF burden and of days in sinus rhythm

AF burden was estimated as the number of ECGs showing AF divided by the total number of ECGs as primary analysis.^8^ As a sensitivity analysis, rhythms were assigned to every day of the monitoring period by forward-filling days without an ECG if the rhythm of the two adjacent ECGs was the same (AF or sinus rhythm). This reflects the protocol requesting telemetric ECG transmission upon symptoms suggestive of AF. A change in rhythm-control therapy—cardioversion, catheter ablation, change in antiarrhythmic drugs, or a physician-reported therapy change during triggered follow-up visits—was treated like an AF ECG. This interpolated dataset was used to calculate days in sinus rhythm divided by all monitoring days for sensitivity analyses.

We performed modelling of rhythm monitoring using weekly or twice weekly short-term ECG recordings to estimate AF burden and compared the AF burden results to the true AF burden (see Supplementary Figs. S6–S10). The AF burden estimates used in this analysis are close to the true AF burden for patients with a high AF burden (Supplementary Fig. S8). Patients with a low AF burden (1–5%) show more variable true AF burdens, especially when only one ECG is transmitted every two weeks (variation between estimated AF burden and true AF burden. Important for this analysis, the assignment to AF burden quartiles was rarely affected by these variations (Supplementary Fig. S10).

### Outcomes

The primary outcome of EAST-AFNET 4 and this analysis was a composite of cardiovascular death, stroke, or unplanned hospitalization for heart failure or acute coronary syndrome. The primary safety outcome was a composite of death from any cause, stroke, or prespecified serious adverse events of special interest capturing complications of rhythm-control therapy. Secondary outcomes included sinus rhythm at 12 and 24 months assessed via regular, singular ECGs, AF recurrence and quality of life assessed via EQ-5D and modified EHRA scores. Rhythm at 12 months was assessed during the 12-months follow-up visit scheduled between 10 and 14 months after randomization, around the start of the landmark period. Secondary endpoints were analysed in a 60-times multiple imputed dataset, utilizing multiple imputation by chained equations (MICE) as previously described.^12^^,^^19^ Motivation was defined as days between randomization and first patient-operated ECG. Adherence was defined as percentage of weeks with one or more patient-operated ECG.

### Statistics

**Fig. 1:**
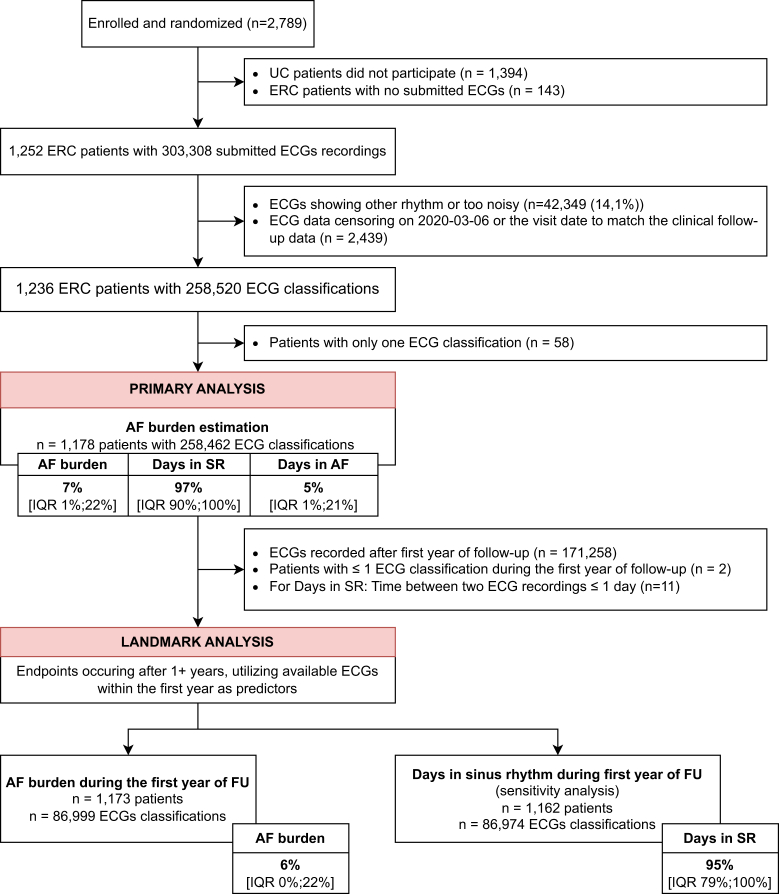
**CONSORT diagram.** Patients enrolled in EAST-AFNET 4 and randomized to early rhythm control were asked to submit telemetric electrocardiograms (ECGs) twice per week. All ECGs were retrieved and analysed employing supervised artificial intelligence in a core lab. The primary analysis population included all patients of which at least two ECGs were available and classifiable for either atrial fibrillation (AF) or sinus rhythm (SR). The *landmark analysis population* included all of those patients, that had more than one ECG during the first year of follow-up until the landmark timepoint at 12 months. Abbreviations: AF = Atrial fibrillation; ECG = Electrocardiogram; ERC = Early rhythm-control; FU = Follow-up; IQR = Interquartile range; SR = Sinus rhythm; UC = Usual Care.

All analyses were performed as randomised in the final, locked data set. Analyses estimating AF burden were performed in all available ECGs only excluded noisy and other-classified ECGs for all burden analyses. The patient population for the *landmark analysis* included all patients randomised to early rhythm control who transmitted at least two telemetric ECGs during the first 12 months of follow-up and who were alive without a primary outcome at 12 months (Fig. 1). ECGs transmitted after the end of the follow-up period were excluded. AF burden in the first year of follow-up was used to assign each patient to an AF burden quartile. Outcomes were compared between quartiles in the 4.1 years of follow-up time after the first year (landmark time point). Patients randomised to usual care served as an external reference. The landmark analysis for the time-to event outcomes employed a Cox proportional hazards model adjusted for age, sex, body mass index (BMI), diastolic blood pressure, heart failure and a frailty-term for the study centre. Analogous to the analysis in the primary paper,^12^ Aalen-Johansen cumulative incidence curves are presented due to the competing events. For the sensitivity analyses, the model was extended to include the type of atrial fibrillation or the presence of SR at baseline. In a further sensitivity analysis, AF burden was estimated based on days in sinus rhythm instead the percentage of ECGs in AF. Analogous to the analysis in the primary paper, the secondary endpoints were analysed using baseline-adjusted mixed linear or logistic regression models and the respective predictor. Locally estimated scatterplot smoothing (LOESS) was utilized to visualize the primary outcome per AF burden as a continuous parameter.

### Role of funding source

The funders did not influence this analysis and were not involved in the decision to publish.

## Results

### AF burden estimation using AI-classified patient-operated short-term telemetric ECGs

**Fig. 2:**
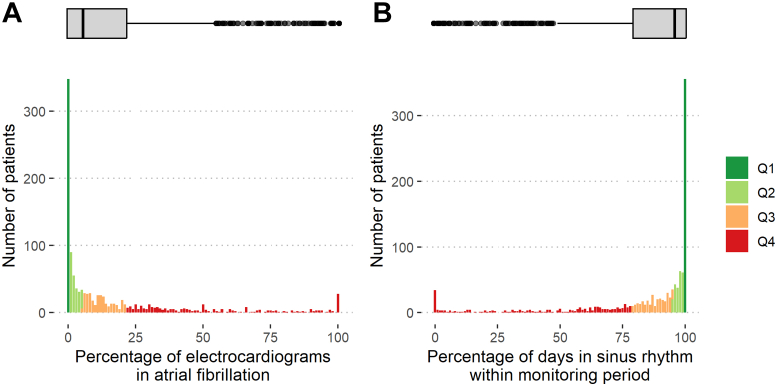
**Distribution of atrial fibrillation (AF) burden (A) and days in sinus rhythm (B) on rhythm-control therapy in the first year of follow-up in the EAST-AFNET 4 trial.** A: AF burden in patients with early rhythm-control within the first year after randomization. B: Number of days in sinus rhythm in the interpolated dataset within the first year after randomization. AF burden was calculated as the number of ECGs in AF divided by the total number of available ECGs in the monitoring period and is displayed in percent (0–100) on the x axis. Similarly, days in sinus rhythm was calculated as the number of days with an ECG in sinus rhythm divided by the monitored time. The height of each column (y-axis) is proportional to the number of patients with a given AF burden or number of days in sinus rhythm. The box plot on top of the histogram shows the distribution of AF burden (A) and days in sinus rhythm (B) in a box-whisker plot. The thick lines indicate the median, boxes illustrate the interquartile range. Abbreviations: AF = Atrial Fibrillation; ECG = electrocardiogram; FU = follow-up; SR = sinus rhythm. Colors and labels refer to AF quartiles, so that patients in Q4 and displayed in red are in the highest AF quartile, and therefore in the lowest SR quartile.

Between July 28, 2011, and December 30, 2016, 1394 participants were randomised to early rhythm-control. Participants transmitted 304,089 telemetric ECGs. These were classified as AF or sinus rhythm (Fig. 1) using the trained algorithm with robust performance metrics (AF classification F1 = 0.903; Supplementary Fig. S1 and Table S1). Each patient transmitted a median of 122 (interquartile range IQR [32; 291]) ECGs over a median of 1095 [IQR 472; 1661] days of follow-up at a median of 1 [IQR 1; 2] per week. Motivation was 3 [IQR 0; 9] days; adherence was 68% [IQR 44%; 88%]; adherence and motivation were correlated with each other (Supplementary Fig. S2). Utilizing all 258,676 ECGs over the full monitoring period, AF burden, defined as ECGs in AF divided by analysable ECGs, was 7% [IQR 1%; 22%] (Fig. 2). Patients spent 5% [IQR 1%; 21%] of monitoring days and 3% [IQR 0%; 10%] of total follow-up days in AF. Days in sinus rhythm were interpolated at 95% [IQR 79%; 99%] of all monitoring days which correlates to 97% [IQR 90%; 100%] of the total follow-up time.

The *landmark analysis* primary analysis population consisted of 1178/1394 patients who transmitted at least 2 ECGs until and were alive without a primary outcome at 12 months of follow-up. The median AF burden in the first year of follow-up in this landmark population was 6% [IQR 0%; 22%]. Patients were 95% [IQR 79%; 100%] of the time in sinus rhythm. Age, sex, and number of comorbidities estimates by CHA_2_DS_2_-VA scores were comparable across AF burden quartiles (Table 1). Diastolic blood pressure was higher and stable heart failure was more prevalent in quartiles Q3 and Q4 by univariate comparison. Adherence to telemetric ECG transmission did not affect AF burden (Supplementary Fig. S3). Initial choice of rhythm-control therapy (none, AF ablation, or antiarrhythmic medication) was not different between AF burden quartiles. Persistent AF at baseline was more common in patients in the higher AF burden quartiles and sinus rhythm at baseline was associated with a lower AF burden in the first year of follow-up (Supplementary Fig. S4).

**Table 1:** Demographic and clinical characteristics of patients at baseline.

AF burden quartile, %	Overall	Quartile groups stratified by proportion of ECGs in AF during the first year of follow-up				p-value^a^
		Q1 [0,0]	Q2 [0,5.8]	Q3 [5.8,21.9]	Q4 [21.9100]	
N^b^	1173	348	240	292	293	
Age (Mean ± SD)	70 ± 8	70 ± 9	70 ± 8	69 ± 8	71 ± 8	0.13
Median (Q1, Q3)	71 (65, 76)	71 (64, 76)	71 (65, 75)	70 (65, 75)	72 (67, 76)	
Female sex	530/1173 (45%)	167/348 (48%)	123/240 (51%)	116/292 (40%)	124/293 (42%)	0.059
Body mass index (kg/m^2^, Mean ± SD)	29.3 ± 5.4	29.4 ± 5.3	29.0 ± 5.4	28.9 ± 5.1	29.8 ± 5.7	0.13
Median (Q1, Q3)	28.6 (25.6, 32.1)	28.6 (26.0, 31.5)	28.2 (25.2, 32.3)	28.2 (25.5, 31.3)	29.0 (25.9, 33.1)	
AF type						<0.001
First episode	445/1173 (38%)	161/348 (46%)	96/240 (40%)	96/292 (33%)	92/293 (31%)	
Paroxysmal	431/1173 (37%)	146/348 (42%)	94/240 (39%)	116/292 (40%)	75/293 (26%)	
Persistent or long-standing persistent	297/1173 (25%)	41/348 (12%)	50/240 (21%)	80/292 (27%)	126/293 (43%)	
Sinus rhythm at baseline	654/1173 (56%)	265/348 (76%)	162/240 (68%)	135/292 (46%)	92/293 (31%)	<0.001
Median days since first AF diagnosis (Median (Q1, Q3))	37 (6, 111)	25 (5, 82)	33 (6, 101)	37 (7, 127)	50 (13, 125)	<0.001
Absence of atrial fibrillation symptoms	342/1108 (31%)	106/328 (32%)	58/229 (25%)	89/274 (32%)	89/277 (32%)	0.24
Previous pharmacological or electrical cardioversion	459/1150 (40%)	146/346 (42%)	90/237 (38%)	117/287 (41%)	106/280 (38%)	0.83
Previous stroke or transient ischemic attack	150/1173 (13%)	43/348 (12%)	28/240 (12%)	36/292 (12%)	43/293 (15%)	0.80
At least mild cognitive impairment	487/1132 (43%)	129/333 (39%)	96/233 (41%)	131/282 (46%)	131/284 (46%)	0.24
**Left atrial size (mm)**	44 ± 8	42 ± 7	43 ± 9	44 ± 9	45 ± 8	<0.001
Arterial hypertension	1036/1173 (88%)	313/348 (90%)	213/240 (89%)	253/292 (87%)	257/293 (88%)	0.70
Systolic blood pressure [mmHg, Mean ± SD]	137 ± 19	136 ± 19	139 ± 20	136 ± 21	138 ± 18	0.30
Systolic blood pressure [mmHg, Mean ± SD]	137 ± 19	136 ± 19	139 ± 20	136 ± 21	138 ± 18	0.30
Diastolic blood pressure [mmHg, Mean ± SD]	81 ± 12	79 ± 12	81 ± 12	82 ± 12	83 ± 12	<0.001
Diabetes treated with diet, medication or insulin, n (%)						0.83
No	900/1173 (77%)	260/348 (75%)	192/240 (80%)	223/292 (76%)	225/293 (77%)	
Yes, treated with diet	54/1173 (4.6%)	17/348 (4.9%)	11/240 (4.6%)	12/292 (4.1%)	14/293 (4.8%)	
Yes, treated with medication or insulin	219/1173 (19%)	71/348 (20%)	37/240 (15%)	57/292 (20%)	54/293 (18%)	
Stable heart failure	323/1173 (28%)	87/348 (25%)	55/240 (23%)	94/292 (32%)	87/293 (30%)	0.015
Left ventricular ejection fraction (%)	59 ± 9	61 ± 9	60 ± 9	58 ± 9	58 ± 10	<0.001
CHA_2_DS_2_-VA score (Mean ± SD)	2.84 ± 0.123	2.88 ± 1.29	2.68 ± 1.12	2.82 ± 1.23	2.96 ± 1.35	0.21
Median (Q1, Q3)	3 (2′, 4)	3 (2, 4)	2 (2, 3)	3 (2, 4)	3 (2, 4)	
Valvular heart disease	514/1173 (44%)	130/348 (37%)	102/240 (43%)	144/292 (49%)	138/293 (47%)	0.078
Chronic kidney disease of MDRF stage 3 or 4	143/1173 (12%)	45/348 (13%)	32/240 (13%)	35/292 (12%)	31/293 (11%)	0.76
Medication at discharge						
Oral anticoagulation with NOAC or VKA	1074/1173 (92%)	307/348 (88%)	217/240 (90%)	269/292 (92%)	281/293 (96%)	0.035
Digoxin or digitoxin	41/1173 (3.5%)	5/348 (1.4%)	4/240 (1.7%)	13/292 (4.5%)	19/293 (6.5%)	0.030
Beta blockers	901/1173 (77%)	262/348 (75%)	186/240 (78%)	233/292 (80%)	220/293 (75%)	0.58
ACE inhibitors or angiotensin II receptor blocker	804/1173 (69%)	238/348 (68%)	172/240 (72%)	191/292 (65%)	203/293 (69%)	0.47
Mineralocorticoid receptor antagonist	75/1173 (6.4%)	22/348 (6.3%)	14/240 (5.8%)	18/292 (6.2%)	21/293 (7.2%)	0.92
Diuretic	457/1173 (39%)	125/348 (36%)	94/240 (39%)	109/292 (37%)	129/293 (44%)	0.27
Statin	529/1173 (45%)	163/348 (47%)	95/240 (40%)	140/292 (48%)	131/293 (45%)	0.20
Platelet inhibitor	183/1173 (16%)	70/348 (20%)	35/240 (15%)	44/292 (15%)	34/293 (12%)	0.027
Planned therapy for rhythm-control at baseline						0.76
AAD	1028/1173 (87.64%)	311/348 (89.37%)	213/240 (88.75%)	254/292 (86.99%)	250/293 (85.32%)	
Ablation	92/1173 (7.84%)	23/348 (6.61%)	16/240 (6.67%)	24/292 (8.22%)	29/293 (9.90%)	
None	53/1173 (4.52%)	14/348 (4.02%)	11/240 (4.58%)	14/292 (4.79%)	14/293 (4.78%)	

Characteristics are shown for the landmark study population and for each AF burden quartile. Absolute counts are shown with relation to the underlying population as well as a percentage; scalars are shown aggregated as median and interquartile ranges or mean with standard deviations depending on data distributions. p values refer to univariate comparisons of differences between AF burden quartiles. Test selection was based on data distributions. Due to the high number of patients without AF on any ECG, the first quartile contains more patients, and the second quartile contains fewer patients than expected.

IQR Interquartile range; ng/L nanograms per litre; Q1–Q4 AF burden quartile 1–4; SD Standard deviation.

Mean (SD) or Frequency with no./total no (%).

a p-values resulting from mixed linear or logistic regression models and Analysis of Deviance Table (Type II Wald chisquare tests). Nominal variables were tested with Pearson’s Chi-squared test.

b 348 patients hat an AF burden of 0%. They were all assigned to the lowest AF burden quartile (Q1), reducing the number of patients in Q2 to 240 patients.

### Effect of AF burden on the primary outcome

**Fig. 3:**
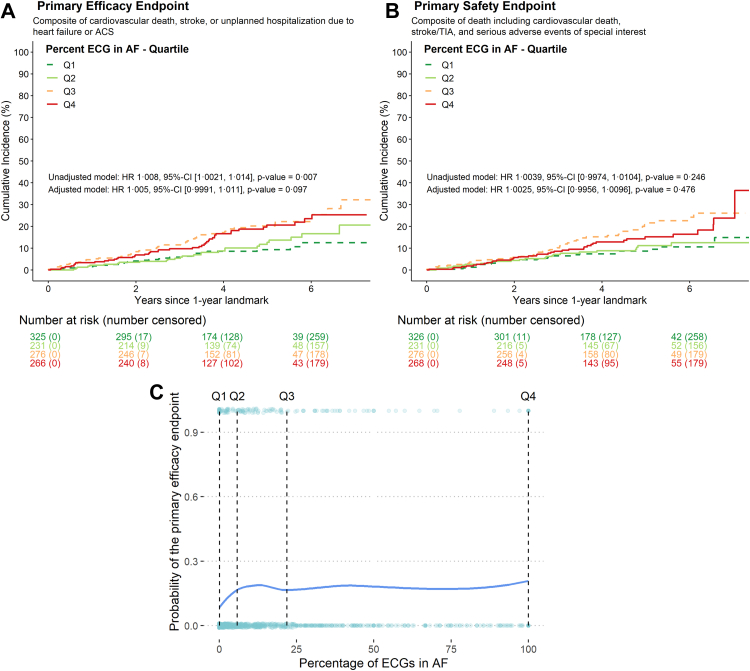
**Aalen-Johansen cumulative-incidence curves by atrial fibrillation (AF) burden quartile for the primary efficacy and safety outcomes in EAST-AFNET 4.** AF burden was estimated by repeated patient-operated ECGs. Numbers give the AF burden percentage boundaries in the four quartiles. The uneven distribution of patients across quartiles is due to the high number of patients with an AF burden of 0%. Starting point of this landmark analysis is the 12-months follow-up. The first year of follow-up was used to determine AF burden. **A:** Incidence rates of the composite primary efficacy outcome by AF burden quartiles in EAST-AFNET 4 patients randomised to early rhythm-control. The primary outcome was a composite of cardiovascular death, stroke, or unplanned hospitalisation for heart failure or acute coronary syndrome. The estimated annualized event rates in the higher AF burden quartiles (Q3: 4.8/100 patient-years; Q4: 4.2/100 patient-years) are quite close to the estimated event rates in patients randomized to usual care (4.5/100 patient-years), while the estimated annualized event rates in Q1 and Q2 were much lower (2 and 2.6/100 patient-years). **B:** Incidence rates of the composite primary safety outcome by AF burden quartiles in EAST-AFNET 4 patients randomised to early rhythm-control. The primary safety outcome was a composite of death from any cause, stroke, or prespecified serious adverse events of special interest capturing complications of rhythm-control therapy. **C:** Probability for each primary efficacy outcome event (y-axis), a composite of cardiovascular death, stroke, or unplanned hospitalization for heart failure or acute coronary syndrome, was plotted continuous utilizing locally weighted scatterplot smoothing (LOESS) for AF burden (x-axis). Dashed lines mark the upper/right boundaries of each AF burden quartile. Abbreviations: AF = Atrial fibrillation; ECG = Electrocardiogram; Q = Quartile Abbreviations: AF = Atrial fibrillation; ECG = Electrocardiogram; Q = Quartile.

In the primary analysis, AF burden was associated with an increased risk of the primary outcome (hazard ratio (HR) per 1 percent increase 1.01 (95% confidence interval (CI): 1.002, 1.014, p = 0.007; Fig. 3). Patients with a low AF burden (Q1, Q2) in the first year of follow-up had low rates of the primary outcome, a composite of cardiovascular death, stroke, or unplanned hospitalisation for heart failure or acute coronary syndrome (Q1: 2.0/100 patient-years; Q2: 2.6/100 patient-years) in the 4.1 years of follow-up. Patients with a high AF burden during the first year of follow-up had higher event rates (Q3: 4.8/100 patient-years; Q4: 4.2/100 patient-years, Fig. 3) that were comparable to event rates in patients randomised to usual care (4.5/100 patient-years, Table 2). When adjusted for age, sex, body mass index, diastolic blood pressure, and heart failure, the attenuated effect was comparable (HR 1.01 (95% CI: 0.9991, 1.011), p = 0.097; Supplementary Fig. S5 and Table S4). When days in sinus rhythm were used to estimate the effect of AF burden in the interpolated dataset (sensitivity analysis), a higher proportion of days in sinus rhythm was linked to fewer cardiovascular events (HR per 1 percent increase 0.991 (95%-CI: 0.986, 0.997), p = 0.002). When adjusted for the same clinical confounders, days in sinus rhythm remained a relevant predictor (HR 0.994 (95%-CI: 0.988, 0.999), p = 0.028, Supplementary Table S2). Further sensitivity analyses adjusted for rhythm at baseline were performed to explore potential unmeasured confounding: in a simple model utilizing AF burden quartiles and sinus rhythm at baseline, both higher AF burden quartiles and sinus rhythm at baseline were independently associated with the primary outcome. The fully adjusted model including clinical confounders confirmed an association of AF burden quartiles with the primary outcome (Supplementary Table S3). A correlation between AF burden and adherence was not observed (Supplementary Fig. S3) which reduces risk of unmeasured confounding by adherence to the monitoring protocol.

**Table 2:** Primary and secondary outcomes by AF burden quartiles in landmark analysis.

	Stratified by proportion of ECGs in AF during the first year of follow-up				Usual care
	Q1 [0,0]	Q2 [0,5.8]	Q3 [5.8,21.9]	Q4 [21.9100]	
First primary efficacy endpoint, events/person-years (incidence/100 person-yr)	27/1321 (2)	27/1027 (2.6)	54/1131 (4.8)	45/1075 (4.2)	228/5013 (4.5)
Components, events/person-years (incidence/100 person-yr)					
Death from cardiovascular causes	8/1405 (0.6)	4/1104 (0.4)	15/1273 (1.2)	13/1199 (1.1)	73/5630 (1.3)
Stroke	2/1383 (0.1)	4/1097 (0.36)	12/1239 (1)	8/1180 (0.7)	47/5505 (0.9)
Hospitalization with worsening of heart failure	14/1362 (1)	14/1070 (1.3)	30/1195 (2.5)	27/1114 (2.4)	126/5223 (2.4)
Hospitalization with acute coronary syndrome	12/1373 (0.9)	8/1066 (0.8)	10/1241 (0.8)	7/1164 (0.6)	46/5468 (0.8)
Secondary outcomes at 12 months					
Sinus rhythm at 12 months	288/304 (94.7%)	221/228 (96.9%)	250/275 (90.9%)	161/262 (61.5%)	788/1203 (65.5%)
Secondary outcomes at 24 months					
Sinus rhythm at 24 months	70/292 (92.5%)	201/217 (92.6%)	225/261 (86.2%)	148/248 (59.7%)	687/1135 (60.5%)
mEHRA III or IV	15/301 (5.0%)	7/222 (3.2%)	15/266 (5.6%)	6/253 (2.4%)	35/1171 (3.0%)
Change in EQ-5D score	1.02 ± 17	0.81 ± 19	−1.28 ± 21.5	−1.81 ± 23.4	−2.73 ± 22.3
Other					
Recurrent atrial fibrillation between days 366 and end of follow-up	137/341 (40.2%)	169/236 (71.6%)	231/291 (79.4%)	183/284 (64.4%)	–

Event rates are displayed as number of events per person-years of follow-up and the corresponding percentage. Changes in EQ-5D score are display as signed score changes and standard deviations.

AF Atrial fibrillation; ECG electrocardiogram; mEHRA Modified European Heart Rhythm Association Score.

### Effect of AF burden on the safety outcome

The primary safety outcome, a composite of death, stroke, and serious adverse events related to rhythm-control therapy, was neither associated with AF burden (HR 1.004 (95%-CI: 0.997, 1.01), p = 0.246) nor days in sinus rhythm (HR 0.996 (95%-CI: 0.99, 1.002), p = 0.189, Supplementary Table S5, Fig. 3). Sinus rhythm at baseline was associated with fewer safety events, driven by fewer deaths (Supplementary Table S6).

### Effect of AF burden on secondary outcomes

AF recurrence occurred in 968/1178 (82%) patients. Time to first AF recurrence was 29 [IQR 5; 154] days. When a conventional blanking and therapy stabilization period of 90 days after randomization was excluded from analysis, 860/1074 patients (80%) had recurrent AF with a median time to recurrent AF of 162 [IQR 106; 418] days. Patients with recurrent AF within 91–365 days of follow-up had an increased risk for a cardiovascular event in a repeated adjusted landmark analysis (hazard ratio per standard deviation 1.6 (95%-CI: 1.11, 2.31), p = 0.012). In multivariable logistic regression analysis for secondary endpoints, sinus rhythm at 12 and 24 months were more common in patients with a low AF burden in the first year of follow-up (sinus rhythm at 12 months: OR 0.97 (95%-CI: 0.96, 0.97), p < 0.001; sinus rhythm at 24 months: OR 0.97 (95%-CI: 0.97, 0.98), p = 0.009). Change in quality of life as assessed via EQ-5D at 24 months was lower in patients with increasing AF burden (adjusted mean difference -0.07 (95%-CI: −0.12, −0.02), p = 0.007). Higher modified EHRA scores (III or IV) were not different with higher AF burden (OR 0.995, 95%-CI: 0.98, 1.01, p = 0.534), probably reflecting the protocol-mandated therapy change upon symptomatic AF, leading to differences in AF burden quartiles of up to 12% in patients with short AF episodes and a low AF burden (Supplementary Fig. S9). In the population analysed here, quartile assignment was highly accurate (variation <1%–3%, Supplementary Fig. S10).

### Comparison of estimated AF burden using regular short-term ECGs and true AF burden

Modelling of the estimated AF burden using regular weekly, twice-weekly, or once every two weeks short-term ECGs, based on generated data sets reflecting the estimated AF burden distribution observed in this study and containing AF patterns with short, medium-length, and longer, persistent episodes consistent with the AF patterns in EAST-AFNET 4^20^ (Supplementary Figs. S6 and S7) found that estimated AF burden correlated well with true AF burden (Supplementary Fig. S8). The difference between estimated AF burden and true AF burden is lowest with twice-weekly ECGs. At high estimated AF burdens (50%–100% AF burden), the variation is only a few percent. At low AF burdens (0%–20% AF burden), the difference is low for patients with long episodes or persistent AF (variation a few percent, Supplementary Fig. S8B and C). The difference between estimated AF burden and true AF burden is higher with short episodes (Supplementary Fig. S8A) and increases with fewer short-term ECGs (right panels in Supplementary Fig. S8, variation 5%–15%) The assignment of patients to AF burden quartiles is only slightly affected, with 75% of patients in the same AF burden quartile in all scenarios (Supplementary Fig. S9). Most differences in assignment to quartiles occur between Q1 and Q2 (both below the median, Supplementary Fig. S10).

## Discussion

This pre-specified post-hoc analysis of the EAST-AFNET 4 trial shows that estimated AF using AI-enabled classification of intermittent, weekly short-term patient-operated ECGs, is associated with cardiovascular events on early rhythm control. A low AF burden (<below the median estimated as 6% in this analysis) in the first year of therapy is associated with low event rates during subsequent follow-up. A high AF burden estimate is associated with higher cardiovascular event rates. Event rates observed in patients with a high estimated AF burden on early rhythm control therapy were comparable to event rates observed in usual care in EAST-AFNET 4. Together, these findings suggest that estimated AF burden on therapy can modulate the effectiveness of rhythm control therapy. Pending external validation and further probing of residual confounding, these results call for integration of AF burden into clinical care and suggest the possibility that patient-operated rhythm recording could be used to estimate AF burden for this purpose.

The stroke-reducing effect of early rhythm-control therapy in EAST-AFNET 4^12^ and of dronedarone in the earlier ATHENA trial^15^ revived the interest in rhythm control therapy and its potential effect on outcomes such as stroke, cardiovascular death, or heart failure events. This analysis suggests that a low AF burden on early rhythm control mediates improved outcomes in EAST-AFNET 4 (Fig. 2). In the two patient quartiles with a low AF burden on early rhythm-control (Q1 and Q2, Table 2) there were only 2 and 4 strokes (event rate <0.5%/100 person-years) while there were 20 strokes in patients in the higher AF burden quartiles (Q3, Q4, Table 2). The low event rate in the low AF burden quartiles, including few strokes, also suggests that a small residual AF burden may not affect stroke risk substantially, supporting a possible “floor effect” on cardiovascular events with very low AF burden^19^ that may also be present after AF ablation.^21^ The concept of withdrawing anticoagulation after AF ablation, justified by the association between very low AF burden and low rate of stroke, is currently tested in the OCEAN trial (NCT02168829). Two moderately-sized controlled trials suggest that AF burden reduction by AF ablation reduces heart failure events.^7^^,^^22^ Consistent with these results, the two low AF burden quartiles in this analysis showed very few heart failure events (Table 2), suggesting that AF burden reduction could be beneficial in patients with AF and heart failure with preserved ejection fraction. Within the limitations of a LOESS analysis, more effective reductions of AF burden may further reduce event rates (Supplementary Fig. S4). The results were consistent in sensitivity analyses using days in sinus rhythm and AF burden as continuous parameters. The results suggest that AF burden estimates can differentiate effective and ineffective rhythm-control therapy. Further analyses of AF burden, its reduction by therapy, and its relation to outcomes are needed to substantiate the role of AF burden for outcome reduction and as a potential surrogate outcome. The ongoing EAST STROKE (NCT05293080), CABAHFPEF-DZHK 27 (NCT05508256), and EAST^high^-AFNET 11 (NCT06324188) trials will provide further information on the outcome-reducing effects of early rhythm-control and early AF ablation. Capturing AF burden in those trials would enable further testing of the effects of AF burden reduction on cardiovascular events with early rhythm control therapy.

AF burden was estimated using regular patient-operated short-term telemetric ECG recordings in this analysis. An identical method was used to estimate AF burden in the ANTIPAF-AFNET 2 trial,^23^ obtaining similar AF burden estimates in paroxysmal AF (10%) as implanted devices.^24^ The same method was also used to estimate recurrent AF in the GAPAF-AFNET 1^25^ and in the Flec SL-AFNET 3 trials.^26^ Combining patient-operated short-term ECGs to estimate AF burden appears to provide a meaningful estimate in this analysis (Supplementary Figs. S6–S10). The estimated AF burden is more precise for higher AF burden values and in patients with longer episodes. Also, increasing the frequency of regular short-term ECGs from once weekly to twice weekly increases the precision of the estimate, especially in patients with low AF burdens. Importantly, this analysis only creates one AF burden estimate over the first year of therapy, integrating spontaneous or therapy-induced changes. Longer-term monitoring, ideally 24/7, will be needed to quantify short-term changes in AF burden, especially in patients with short episodes and with a low overall AF burden. The median AF burden estimate in patients in the first year after initiating early rhythm-control was 6% (IQR 0%–22%) in this analysis is within the range or slightly below the estimated AF burden in patients in the drug arms of the MANTRA-PAF^27^ and EARLY-CRYO trials.^28^ In hindsight, the protocolised recommendation in EAST-AFNET 4 to adjust rhythm-control therapy upon documentation of at least two adjacent ECGs with recurrent AF probably focused therapy adjustments on patients with a high AF burden. Lower AF burden estimates appeared to be associated with reduced event rates (Fig. 3C). AF burden estimation based on one year of intermittent, patient-generated ECGs, analysed via AI, yielded outcome-relevant insights. Some AF management questions, e.g., relating rhythm control to reduced heart failure events or symptoms, may need to estimate AF burden over shorter time periods, potentially requiring more intensive rhythm monitoring e.g., using loop recorders or wearable consumer electronics. Furthermore, AF burden estimates using intermittent rhythm monitoring will be imprecise (Supplementary Figs. S6–S10) and may carry systemic errors.^11^ Hence, our results call for further analyses simulating different AF monitoring patterns in existing AF burden data sets and relating them to different outcomes that are relevant for patients.

Time to first recurrence of AF is the most commonly used efficacy outcome for rhythm-control therapies. This outcome was observed in around 80% of patients within a few months of early rhythm-control therapy in this analysis. More than half of the patients with a low AF burden and low cardiovascular event rates showed recurrent AF. The present results, and an earlier report assessing the mediator effect of rhythm at 12 months,^29^ suggest that a singular first recurrence of AF has little effect cardiovascular outcomes, questioning the relevance of this outcome for patients. These considerations and the results of this paper support to consider AF burden not only as an outcome in early drug development, but also as an efficacy outcome for registration of rhythm-control therapies. This will require collection of safety information (Supplementary Table S6).

Our data support the concept that lowering AF burden should be a therapeutic goal in patients with AF as suggested by the recent AHA/ACC/HRS AF guidelines.^9^ This observation needs deeper exploration with respect to relevant time intervals of AF burden data collection, time intervals of AF burden reduction, and finally verification in larger data sets integrating information on cardiovascular events, clinical features, and anticoagulation, as well as rhythm-control therapies.

Integration of AF burden into clinical care will face challenges: AF burden is currently defined heterogenously across devices and rhythm monitoring technologies, and AF burden information is not yet interoperable. The shift from physician-estimated AF patterns to physician-generated or patient-generated AF estimates marks a fundamental transition, calling for robust framework for data validation, standardization, and clinical workflow integration.^30^

Strengths of the analysis include analysis of a clinical trial data set containing adjudicated events with long follow-up (4.1 years after the landmark date) in an international, multi-center, controlled trial, and validated, automated methods to determine AF burden. This enabled a robust assessment of AF burden and trial outcomes.

This analysis has several limitations. First, AF burden was estimated using patient-operated telemetric ECGs. Such an estimate has been used before^23^ and adherence and motivation did not influence AF burden (Supplementary Fig. S2). Nonetheless the present AF burden estimates will be influenced by recording intensity and patient behaviour. Furthermore, the AF burden estimate used here provides an average AF burden over one year of short-term recordings, ignoring time- and therapy-dependent short-term changes in AF burden. The method for recording patient-operated ECGs was timely and effective when the trial was started but has since been discontinued. Modern technology can obtain more frequent recordings via consumer electronics, allowing to collect more data on AF burden per patient with less effort.^10^ Modelling of AF burden estimates using once or twice weekly short-term ECGs over a year compared to true AF burden, assuming a range of AF episode patterns and the distribution of AF burden observed in this study, found that AF burden estimates are comparable to the true AF burden with more variability in patients with a low AF burden. This is a limitation of this method. Despite this variability, the assignment to AF burden quartiles appeared to make few errors, especially in the discrimination between the lower two AF burden quartiles and the upper two AF burden quartiles. Second, telemetric ECGs were only recorded in patients randomised to early rhythm-control, precluding comparison to patients receiving usual care. The usual care group served solely as a calibrator of event rates. Third, the short AF duration prior to randomization (37 days median in the primary analysis population) and the trial design precluded estimation of AF burden prior to therapy initiation. Fourth, the boundaries of the AF burden quartiles are data-driven and may not represent the optimal differentiation based on underlying biology or pathophysiology. To mitigate this, we employed two definitions of AF burden (percentage of ECGs in AF and days in sinus rhythm in an interpolated dataset) for analysis with very similar outcomes and also report the effects of AF burden as a continuous parameter. Further analyses in large data sets with robust AF burden estimation and outcome information are needed to overcome this limitation. Fifth, the landmark analysis creates a potential immortal time bias by excluding patients who died or experienced a primary outcome event in the first year of follow-up. Sixth, monitored time did not allow to discern different AF patterns yielding similar burdens, e.g., comparing patients with a few long episodes to patients with multiple short episodes of AF. More intensive rhythm monitoring using wearables or implanted loop recorders is needed to address this. Seventh, the prespecified analysis did not include formal analyses of components of the primary outcome. These are given as descriptive numbers and incident rates without statistical comparison, as is typical for composite primary outcomes. Eigth, left atrial volume,^31^ atrial strain,^32^^,^^33^ and cardiovascular biomarkers^33^^,^^34^ can predict AF, AF burden, and AF-related outcomes. These parameters were not included in this analysis. Ninth, additional residual confounding may have contributed to the present results. Tenth, the EAST-AFNET 4 trial enrolled a European population. Finding may differ in other ethnicities. Finally, AF burden estimation would benefit from standardization^10^ to advance the field and ensure consistency across future studies.

In summary, AF burden estimation in the first year of early rhythm control therapy based on weekly short-term patient-operated ECGs modulates the effect of early rhythm-control on cardiovascular events in this analysis of EAST-AFNET 4. These hypothesis-generating results suggest that AF burden can modulate the outcome-reducing effect of rhythm control therapy. Important limitations are the possibility of residual confounding and the fact that AF burden estimates were based on one year of weekly short-term recordings. Further research into the possible outcome-modulating effect of AF burden reduction via rhythm-control therapy is warranted.

## Contributors

S.Z., U.S., P.K.: Conceptualization, Software, Formal analysis, Validation, Writing—original draft, Writing—review & editing. K.B.: Conceptualization, Formal analysis, Visualization, Writing—review & editing. J.O.: Writing—original draft, Writing—review & editing, Validation, Formal analysis, Conceptualization. A.J.C., L.F, A.G, L.E., H.J.G.M.C., M.D.L.,C.M., A.M., A.R., R.B.S., P.V., S.W.: Investigation, Resources, Writing—review & editing. A.S.: Review of statistical analysis. E.S., J.H.: Modelling of AF burden estimates, critical revision. Z.H., B.J.M.H: Software, Formal analysis. A.Z.: Formal analysis, Supervision. K.B., A.S. and P.K. had access to and verified the underlying data. S.Z. and U.S. had access to the artificial intelligence-based classification of the telemetric ECG data. All authors have read and agree to the publication of the final manuscript.

## Data sharing statement

Data will be made available on reasonable request (contact: info@kompetenznetz-vorhofflimmern.de). The protocol was approved by the ethics review boards of all institutions involved. All patients participating in the trial provided written informed consent.

## Declaration of interests

S.Z., K.B., Z.H., B.J.M.H., E.S.: Nothing to declare.

U.S. received research grants to institution from EU, Roche, and the Dutch Heart Foundation.

U.S. received consulting fees or honoraria from Roche Diagnostics (Switzerland), YourRhythmics BV, University Lugano, and Johnson & Johnson. U.S. is a member of Data Safety Monitoring Boards/Advisory Boards of Roche, YourRhythmics BV, and EP Solutions and is co-founder and shareholder of YourRhythmics BV, a spinoff company of the University Maastricht.

J.O. received research grants from German Heart Foundation, University of Hamburg, German Federal Ministry of Education and Research, and German Center for Cardiovascular Research. In addition, J.O. acts as Managing Director of the IDM gGmbH, subsidiary of the University Medical Center Hamburg-Eppendorf. J.O. received invitations and travel grants to Abbott-organised electrophysiology summits on mapping.

A.J.C. received consulting fees from Bayer, Daiichi Sankyo, Acesion, InCarda Therapeutics, Abbott, Boston Scientific, Medtronic, Huya Bio, Biosense Webster, and lecture honoraria from Sanofi and Menarini. A.J.C. is a member of Data Safety Monitoring Boards/Advisory Boards of Anthos, AFNET, Johnson and Johnson (all paid), Attune, the British Heart Foundation, and Charité (all unpaid). A.J.C. discloses a leadership or fiduciary role in Drug Safety Research Unit, Arrhythmia Alliance, Atrial Fibrillation Association and European Society of Cardiology (all unpaid).

H.J.G.M.C. received honoraria from Medtronic, Atricure, and support for attending meetings and/or travel from Occidental Cardiology Congress. H.J.G.M.C. is a member of Data Safety Monitoring Boards/Advisory Boards of Decision trial, Abacus trial, EASThigh trial, and ARMGO. H.J.G.M.C. discloses a leadership or fiduciary role in the DZHK-German Centre for Cardiovascular Research.

L.E. received research support from German Heart Foundation, consulting fees from Boston Scientific and lecture fees from various medical companies. L.E. discloses a leadership or fiduciary role in the German Society of Cardiology.

L.F. received institutional research grants by EU 633196 (CATCH-ME), EU 847770 (AFFECT-EU) and EU 965286 (MAESTRIA). British Heart Foundation (AA/18/2/34218), German Center for Cardiovascular Research (DZHK) supported by the German Ministry of Education and Research. In addition, L.F. received institutional research grants from NIHR, Medical Research Council (UK) and DZHK. L.F. received lecture honoraria from Roche (to institution), equipment and analytics to AFNET for AFNET9 (Smart in OAC) from Preventicus, and trial costs to AFNET from Daiichi-Sankyo. L.F. is listed as inventor on two issued patents held by the employing institution (Atrial Fibrillation Therapy WO 2015140571, Markers for Atrial Fibrillation WO 2016012783). L.F. discloses faculty support for attending meetings and/or travel and a leadership or fiduciary role in BHF project grant committee, BHF chair committee visits, AFNET steering committee, and ARVC patient organization.

A.G. received consulting fees from Daiichi Sankyo and payment or honoraria from Daiichi Sankyo, Bayer, Boehringer, Pfizer, Bristol-Meyers Squibb, Boston Scientific and Medtronic.

J.H. discloses a research contract between InCarda Therapeutics an the Medical University of Graz (institution).

M.D.L. received a travel grant from biosense webster and a research grant from Farapulse. In addition, he was supported by the Research Promotion Fund of the Faculty of Medicine (Hamburg, UKE, “Clinician Scientist Program”).

C.M. received research funding from German Center for Cardiovascular Research (DZHK; Promotion of women scientists programme; FKZ 81X3710112), the Deutsche Stiftung für Herzforschung, the Dr. Rolf M. Schwiete Stiftung, NDD, and Loewenstein Medical unrelated to the current work. In addition, C.M. received speaker fees from Edwards, AstraZeneca, Novartis, Boehringer Ingelheim/Lilly, Bayer, Novo Nordiskand support for attending meetings and/or travel from Edwards, AstraZeneca, Novartis, Boehringer Ingelheim/Lilly, Bayer, and Novo Nordisk. C.M. is a member of Data Safety Monitoring Boards/Advisory Boards of Boehringer Ingelheim and Novo Nordisk.

A.M. received consulting fees from Medtronic and Biosense Webster and honoraria from Medtronic, LifeTech, Biosense-Webster, Daiichi Sankyo, Boston Scientific, Haemonetics, Bayer, and Bristol Myers Squibb. In addition, A.M. received travel support from Medtronic, Bayer, Atricure, Abbott, Daiichi Sankyo, Biosense Webster, Boston Scientific, Haemonetics, Pfizer, Bristol Myers Squibb, and LifeTech.

A.R. received a research grant form Medtronic and Else Kröner Fresenius Stiftung. In addition, A.R. received consultant fees from Medtronic, KODEX-EPD, Biosense Webster, Boston scientific, Atricure and travel grants and lecture fees from Medtronic, Cardiofocus, Biosense Webster, Abbott, Boehringer Ingelheim, Philips KODEX-EPD, Ablamap, Bayer, Novartis, Lifetech, Boston Scientific, Atricure, and Lilly. A.R. is a member of Data Safety Monitoring Boards/Advisory Boards of Abbott, Attricure, Boston scientific and discloses a leadership or fiduciary role in the German Society of Cardiology.

R.B.S. has received funding from the European Research Council (ERC) under the European Union’s Horizon 2020 research and innovation programme under the grant agreement No 648131, from the European Union’s Horizon 2020 research and innovation programme under the grant agreement No 847770 (AFFECT-EU) and German Center for Cardiovascular Research (DZHK e.V.) (81Z1710103 and 81Z0710114); German Ministry of Research and Education (BMBF 01ZX1408A) and ERACoSysMed3 (031L0239). Wolfgang Seefried project funding German Heart Foundation. In addition, R.B.S. received lecture fees and advisory board fees from BMS/Pfizer and Bayer outside this work.

A.S. received grants to the institution for statistical analysis from EU Horizon 2020, Biotronik and Adrenomed AG and a grant from AFNET for statistical analysis.

P.V. received consulting fees from Hygeia Hospitals Group HHG.

S.W. received grants from Abbott and Boston Scientific outside the submitted work. In addition, S.W. received consulting fees from Abbott and Boston Scientific, and lecture honoria from Abbott and Boston Scientific, Bristol Myers Squibb and Medtronic.

A.Z. received grants to the institution for statistical analysis from EU Horizon 2020, Biotronik and Adrenomed AG and a grant from AFNET for statistical analysis. A.Z. received personal fees for lectures from Boston Scientific.

P.K. was partially supported by European Union MAESTRIA (grant agreement 965286), British Heart Foundation (AA/18/2/34218), German Center for Cardiovascular Research supported by the German Ministry of Education and Research (DZHK, grant numbers DZHK FKZ 81X2800182, 81Z0710116, and 81Z0710110), German Research Foundation (Ki 509167694), Dutch Heart Foundation (DHF), the Accelerating Clinical Trials funding stream in Canada, and the Else-Kröner-Fresenius Foundation. P.K. received honoraria, travel support and payment for expert testimony from several pharmaceutical and medical device companies in the past, but not in the last five years. P.K. is listed as inventor on two issued patents held by the University Medical Center Hamburg-Eppendorf (Atrial Fibrillation Therapy WO 2015140571, Markers for Atrial Fibrillation WO 2016012783). P.K. discloses a leadership or fiduciary role as speaker of the Board of AFNET, Germany, and as Board member, ESC.
