# Supplementary material for: Estimated atrial fibrillation burden on early rhythm-control and cardiovascular events in the EAST-AFNET 4 trial
Source: eClinicalMedicine. 2025 Sep 1;88:103457. doi: 10.1016/j.eclinm.2025.103457 (PMC12572810; doi:10.1016/j.eclinm.2025.103457)
Supplement: Supplementary Figs. S1–S10 and Table S1–S6 [file mmc1.pdf]

Atrial fibrillation burden on therapy modifies the outcome-reducing effect of early rhythm-control therapy: EAST-AFNET 4 rhythm monitoring study.

Supplementary Information

Short Title: AF burden on rhythm-control and outcomes in EAST-AFNET 4

Stef Zeemering PhD[1*], Katrin Borof[2*]; Ulrich Schotten, MD, PhD[1,3,4]; Julius Obergassel, MD[2,5,6]; A John Camm, MD, PhD[7]; Harry J G M Crijns, MD, PhD[3]; Lars Eckardt, MD[4,8]; Larissa Fabritz, MD[2,4,5,6,9]; Andreas Goette, MD[4,10,11]; Zarina Habibi, MD[3], Jordi Heijmans, PhD[3,12], Ben JM Hermans, PhD[1], Marc D Lemoine, MD[2,5,6]; Christina Magnussen, MD[2,6,12]; Andreas Metzner, MD[2,6]; Andreas Rillig, MD[2,6]; Renate B. Schnabel, MD, MSc[2,4,6]; Eva Schuijt, MSc[1];Anna Suling M. Sc[16], Panos Vardas, MD[13,14]; Stephan Willems, MD[4,15], Antonia Zapf, PhD[16]; Paulus Kirchhof, MD[2,4,6,9]

Supplementary Results

ECG annotation. In total 3,587 ECGs (2,443 training/1,144 test) were extracted for manual rhythm annotation. ECGs were labelled as SR (n=1,433/829), AF (360/134), Other (75/18) and Noise (575/163).

Classifier performance. The CNN trained to discriminate noisy ECGs reached a training accuracy of 90.3% and a test accuracy of 92.3%. The SVM rhythm classifier with a quadratic kernel reached an overall test accuracy of 96.0%, with an overall F1-score of 0.795, and an AF F1-score of 0.903. The two-stage procedure led to an overall test accuracy of 89.8%, with an F1-score of 0.766, and an AF F1-score of 0.866. Lower F1-scores were mostly driven by lower performance on ECGs labelled as 'Other', with higher F1-scores for AF and SR classes (Supplemental Table 1).

Classification results. Out of 304,089 available ECGs, 781 (0.3%) were not eligible for analysis due to invalid recording date or failed QRS detection. Out of the remaining 303,308 ECGs, 39,503 (12.9%) were classified as Noise. After exclusion of these ECGs, 263,879 ECGs remained from 1,226 patients. Rhythm classification resulted in SR in 86.6% of ECGs, AF in 12.2%, and Other in 1.2%.

Accuracy of AF burden estimation. To assess the accuracy of estimating AF burden based on the percentage of patient-operated short-term ECGs in AF, an artificial dataset was generated consisting of episodes of AF interspersed with random intervals of SR, chosen to achieve a predefined AF burden. Three AF patterns were generated: short AF episodes (episode duration 120±20 min; Supplemental Figure 6A), long AF episodes (episode duration 720±120 min; Supplemental Figure 6B) and persistent AF (episode duration 10,080±1,440 min; Supplemental Figure 6C) to cover a range of potential AF patterns in the EAST-AFNET 4 population (paroxysmal AF, first diagnosed AF, persistent AF). With each of the patterns a virtual cohort was generated with AF burdens matching the estimated burdens based on Figure

2 (see Supplemental Figure 7). Subsequently, the correlation between the true AF burden and the percentage of ECGs in AF was assessed when a virtual ECG (i.e., rhythm assessment) was performed twice weekly, once weekly, or once every 2 weeks. Supplemental Figure 8 shows that the ECG-based estimation shows a good correlation with the true AF burden and that the correlation becomes more accurate with more frequent virtual ECG recordings. The correlation is strongest for persistent AF (Supplemental Figure 8C), where the long episode duration ensures that most episodes are covered by a recording moment. Finally, we assessed the accuracy of assigning artificial rhythm patterns to one of the four AF burden quartiles (Q1: (0,0]%, Q2: (0,5.8]%, Q3: (5.8,21.9]%, or Q4: (21.9,100]%; Table 1) based on twice weekly, once weekly, or once-every-2-weeks ECG recordings compared to an assignment based on the true AF burden (Supplemental Figure 9). With 26 ECGs (one every 2 weeks), 73%, 74% and 90% of rhythm patterns were assigned to the correct quartile for AF episodes with short duration, long duration or persistent AF, respectively (Supplemental Figure 9). This accuracy rose to 82%, 83% and 97% with once weekly recordings, and rose further to 91%, 90% and 98% with twice weekly recordings (Supplemental Figure 9). For a virtual population comprising 1/3 individuals with short-duration AF episodes, 1/3 individuals with long-duration AF episodes and 1/3 individuals with persistent AF (roughly resembling the division between first-diagnosed AF, paroxysmal AF and persistent AF in the EAST AFNET-4 population) the accuracy for twice weekly, once weekly, or once-every-2-weeks ECG recordings was 92%, 87% and 79%, respectively (Supplemental Figure 10), indicating that the percentage of ECGs in AF represents a reliable estimate of AF burden.,), indicating that the percentage of ECGs in AF represents a reliable estimate of the true AF burden.

Supplementary Tables

Supplemental Table 1: Noise and rhythm classification performance. *Abbreviations: AUC = area under the receiver operating characteristic curve; SR sinus rhythm; AF atrial fibrillation/atrial flutter*

	Accuracy		AUC		F1-score	
Model	Training	Test	Training	Test	Training	Test
Noise	90.3%	92.3%	0.977	0.964	0.849	0.855
Rhythm	94.5%	96.0%	0.980	0.986	0.777	0.795
SR	96.1%	96.9%	0.971	0.984	0.975	0.982
AF	96.8%	97.4%	0.982	0.983	0.920	0.903
Other	96.1%	97.8%	0.763	0.918	0.438	0.500
Two-stage	-	89.8%	-	-	-	0.766
Noise	-	92.7%	-	-	-	0.772
SR	-	92.3%	-	-	-	0.945
AF	-	96.9%	-	-	-	0.866
Other	-	97.7%	-	-	-	0.482

Supplemental Table 2: Sensitivity analysis for the first primary efficacy outcome utilizing days in sinus rhythm as the rhythm predictor; univariate and multivariable, adjusted Cox-regression with a landmark at 12 months after randomization. The EAST-AFNET 4 primary efficacy endpoint, a composite of cardiovascular death, stroke, or unplanned hospitalisation with worsening heart failure or acute coronary syndrome, was analyzed using univariate and multivariable, adjusted Cox-regression models. A landmark at 12 months for endpoints was introduced and days in sinus rhythm (SR) within the first year of follow-up before the landmark served as the rhythm predictor. *Abbreviations: AF Atrial fibrillation; CI Confidence interval; ECG electrocardiogram; HR Hazard ratio; SD Standard deviation; SR Sinus rhythm*

	HR	95%-CI	p-value
Univariate model			
Days in SR	0. 991	0.986, 0.997	0.002
Multivariable model			
Days in SR	0.994	0.988, 0.999	0.028
Male sex	1.36	0.98, 1.9	0.071
Age (10 years increase)	1.8	1.42, 2.28	0.708
Body-mass-index	0.99	0.96, 1.03	0.048
Diastolic blood pressure	1.01	1, 1.03	0.033
HF and LVEF<35%	2.78	1.09, 7.09	0.036
HF and LVEF>=35%	1.46	1.03, 2.07	0.125

Supplemental Table 3: Sensitivity analysis for the primary efficacy outcome, adjusted for heart rhythm at baseline; multivariable Cox-regression with a landmark at 12 months after randomization. Atrial fibrillation burden (AF burden) was defined as percentage of electrocardiograms (ECGs) in AF out of all received, analyzable ECGs. AF burden quartiles were used for this analysis which was additionally adjusted for sinus rhythm at baseline. The primary efficacy outcome of EAST-AFNET 4 was a composite of cardiovascular death, stroke, or unplanned hospitalization with worsening heart failure or acute coronary syndrome. ECG *Abbreviations: AF Atrial fibrillation; CI Confidence interval; ECG electrocardiogram; HR Hazard ratio; SD Standard deviation*

	HR (reference 1. quartile)	95%-CI	p-value
AF ECGs / all telemetric ECGs in Q2 (0,5.8]	1.26	0.73, 2.16	0.41
AF ECGs / all telemetric ECGs in Q3 (5.8,21.9]	2.07	1.28, 3.36	0.003
AF ECGs / all telemetric ECGs in Q4 (21.9,100]	1.82	1.90, 3.03	0.022
Sinus rhythm at baseline	0.68	0.47, 0.96	0.031

Supplemental Table 4: Univariate and multivariable Cox-regression with a landmark at 12 months after randomization for the primary efficacy outcome. The primary efficacy outcome of EAST-AFNET 4 was a composite of cardiovascular death, stroke, or unplanned hospitalization with worsening heart failure or acute coronary syndrome. *Abbreviations: AF Atrial fibrillation; CI Confidence interval; ECG electrocardiogram; HR Hazard ratio; SD Standard deviation*

	HR	95%-CI	p-value
Univariate model			
AF ECGs / all telemetric ECGs	1. 01	1.002, 1.01	0.007
Multivariable model			
AF ECGs / all telemetric ECGs	1.01	0.98, 1.01	0.097
Male sex vs female	1.36	0.98, 1.90	0.068
Age (per 10 years increase)	1.78	1.41, 2.24	< 0.001
Body-mass-index	0.995	0.96, 1.03	0.779
Diastolic blood pressure	1.02	1.001, 1.03	0.035
HF and LVEF<35% vs. no HF	2.73	1.07, 6.98	0.036
HF and LVEF>=35% vs. no HF	1.50	1.06, 2.13	0.023

Supplemental Table 5: Safety outcome by AF burden quartile. Event rates are displayed as number of events per person-years of follow-up and the corresponding percentage in percent per 100 person-years. *Abbreviations: AF Atrial fibrillation; ECG electrocardiogram*

	Stratified by proportion of ECGs in AF during the first year of follow-up				Usual care
	**(0,0]**	**(0,5.8]**	**(5.8,21.9]**	**(21.9,100]**	
Safety outcome	27/1670 (1.6%)	23/1281 (1.8%)	50/1441 (3.5%)	38/1413 (2.7%)	171/6737 (2.5%)
Components of the safety outcome, events/person-years (incidence/100 person-yr)					
Death from any cause	22/1741 (1.3)	12/1341 (0.9)	32/1561 (2)	30/1478 (2)	133/6954 (1.9)
Stroke	2/1383 (0.1)	4/1097 (0.36)	12/1239 (1)	8/1180 (0.7)	47/5505 (0.9)
Serious adverse events related to rhythm-control therapy	3/1695 (0.2)	9/1289 (0.7)	14/1476 (0.9)	3/1434 (0.2)	9/6873 (0.1)

Supplemental Table 6: Safety outcome by sinus rhythm at baseline. Probability of the primary safety outcome was analyzed within the overall EAST-AFNET 4 trial population including randomization group and sinus rhythm at baseline as predictors. Second, the probability of the primary safety outcome was analyzed in the landmark analysis population including atrial fibrillation burden quartile and sinus rhythm at baseline as predictors. *Abbreviations: AF Atrial fibrillation; CI Confidence interval; ECG electrocardiogram; HR Hazard ratio; SD Standard deviation*

	HR	95%-CI	p-value
Overall EAST-AFNET 4 trial			
Early rhythm-control	1. 07	0.89, 1.29	0.467
Sinus rhythm at baseline	0.71	0.59, 0.86	0.001
Landmark analysis population of early rhythm-control group			
AF ECGs / all telemetric ECGs in Q2 (0,5.8]	1.15	0.65, 2.02	0.634
AF ECGs / all telemetric ECGs in Q3 (5.8,21.9]	2.24	1.37, 3.64	0.001
AF ECGs / all telemetric ECGs in Q4 (21.9,100]	1.69	0.99, 2.86	0.05
Sinus rhythm at baseline	1.13	0.78, 1.63	0.524

Supplementary Figures

Supplemental Figure 1. Examples of ECGs annotated by independent observers as Sinus rhythm, Atrial fibrillation, Other (e.g. frequent premature ventricular complexes), and an ECG in which rhythm could not be assessed (Noise). Depicted are middle 10-second segments of 30-second signals.

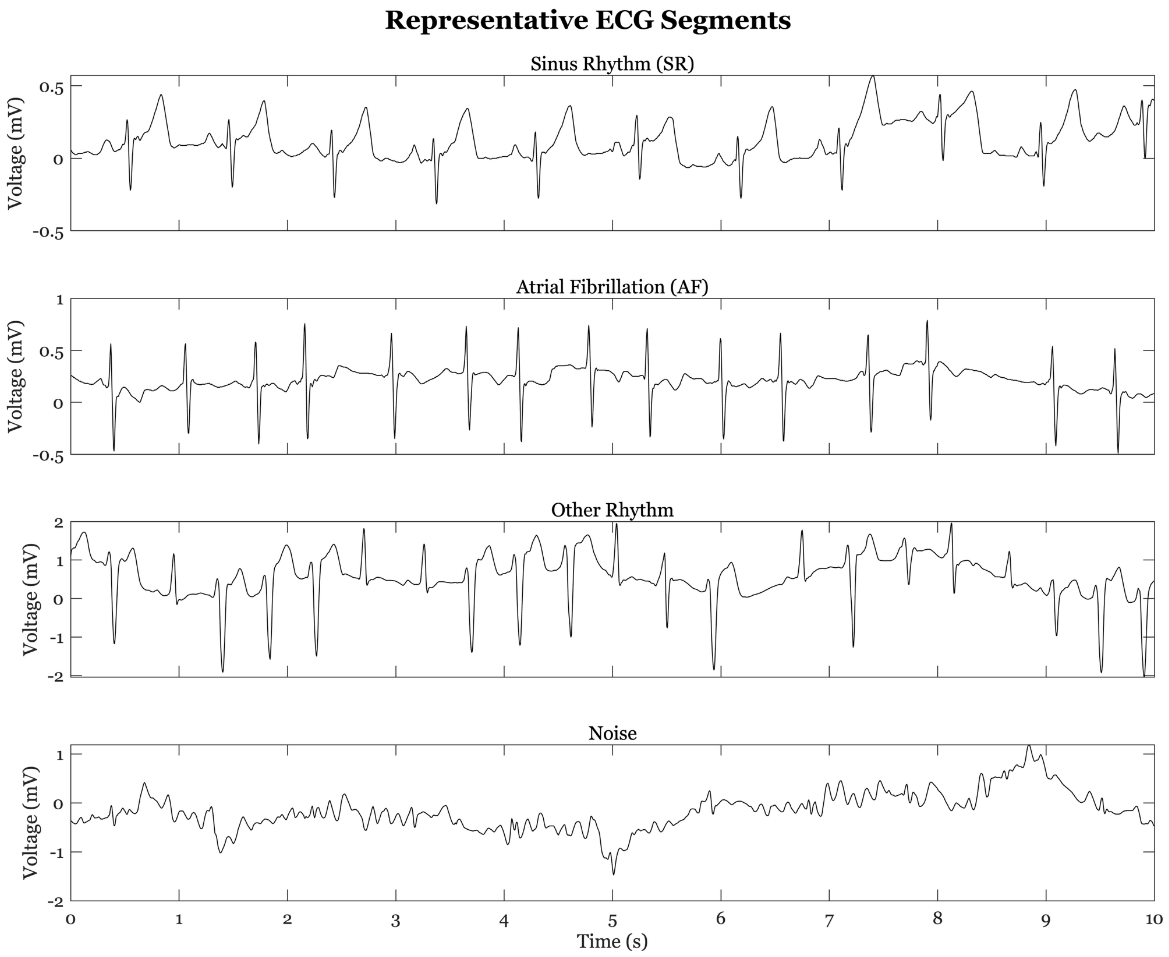

Supplemental Figure 2: Correlation of adherence and motivation. Motivation was defined as days until first submitted telemetric electrocardiogram (ECG); adherence was defined as percentage of weeks with one or more ECG submitted.

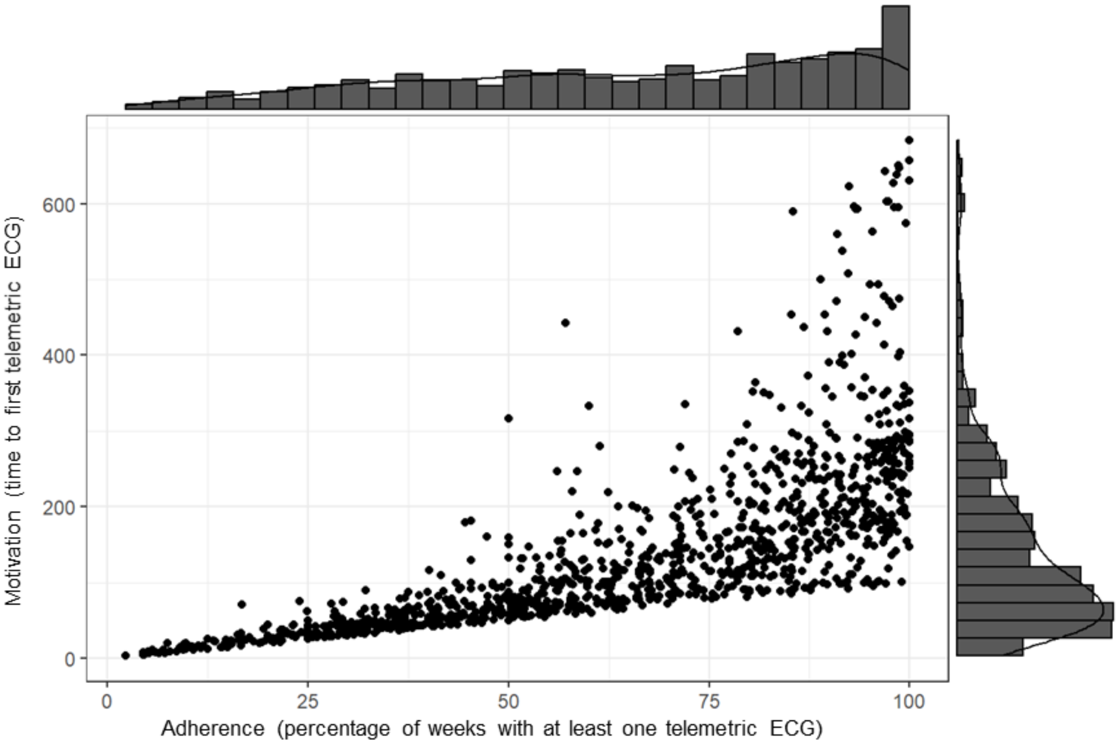

Supplemental Figure 3: Correlation of adherence and atrial fibrillation (AF) burden. Continuous AF burden is plotted over adherence, defined as percentage of weeks with one or more submitted telemetric electrocardiograms (ECG). Locally estimated scatterplot smoothing (LOESS) was utilized to visualize the primary outcome per AF burden as a continuous parameter. *Abbreviations: AF = atrial fibrillation; ECG = electrocardiogram*

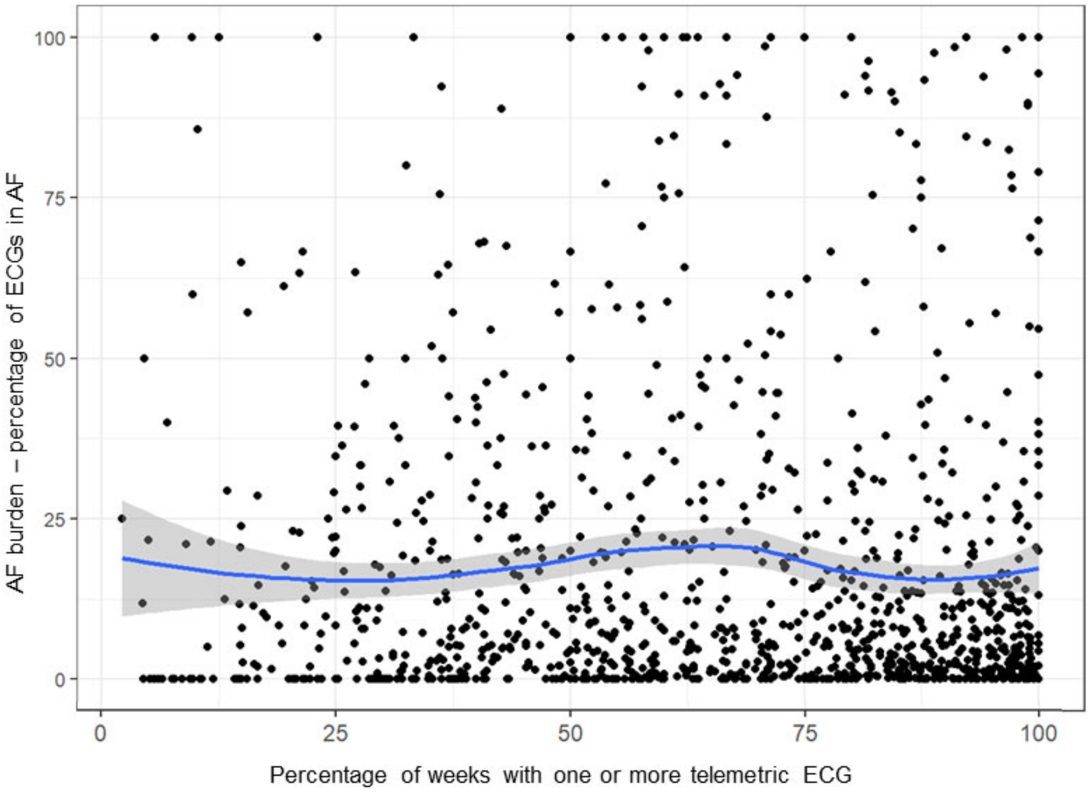

Supplemental Figure 4: Atrial fibrillation (AF) burden by presence of sinus rhythm at baseline. AF burden during the first year of follow-up as defined by percentage of electrocardiograms (ECG) in AF was box-plotted based on presence of sinus rhythm (SR) or other rhythm at the baseline (BL) visit of the EAST-AFNET 4 randomized controlled trial. *Abbreviations: AF = atrial fibrillation; BL = baseline; ECG = electrocardiogram; SR = sinus rhythm*

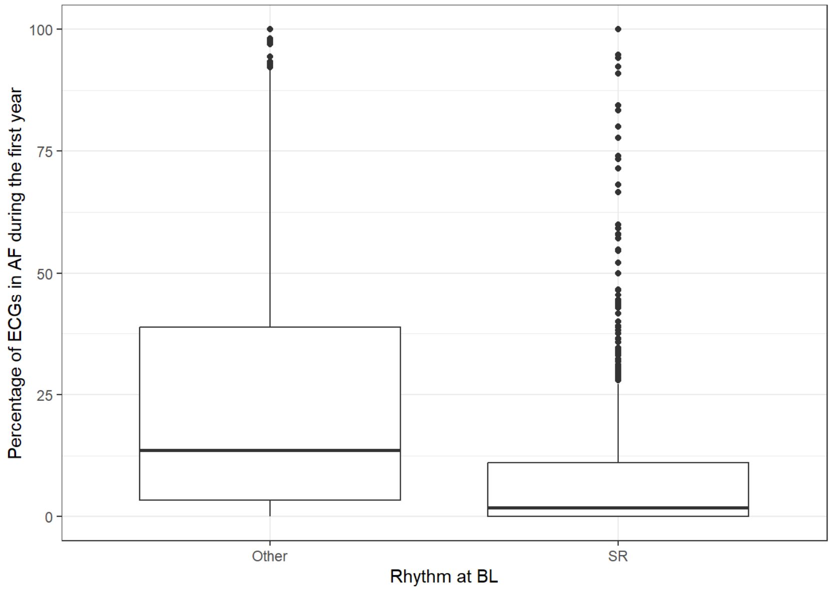

Supplementary Figure 5: Atrial fibrillation (AF) burden and clinical confounders associated with primary outcome events in the 4.1 years of follow-up in the landmark analysis. The forest plot displays hazard ratios per standard deviation (HR per SD) for AF burden, as well as the utilised clinical confounder age (per 10 years increase), body mass index (BMI) and male sex and diastolic blood pressure and heart failure (HF) category by left ventricular ejection fraction (LVEF) below or above 35% as HR. *Abbreviations: BMI = Body Mass Index; HF = Heart Failure; HR = Hazard Ratio; LVEF = Left ventricular ejection fraction; SD = Standard Deviation*

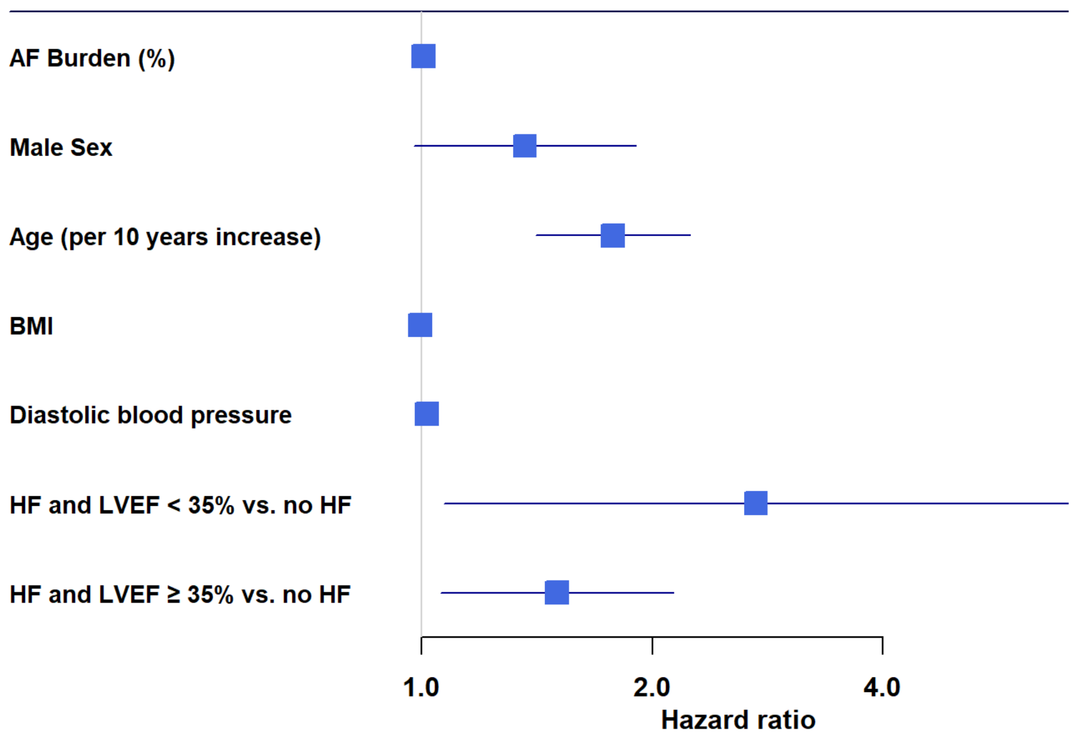

Supplemental Figure 6: Examples of artificially generated AF patterns with low or intermediate AF burden used to evaluate the accuracy of repeated ECG-based assessment of AF burden. A. AF episodes (blue) with short duration (120±20 min) and a burden of 2% (left) or 10% (right) during 365 days of follow-up. B. Episodes with long duration (720±120 min). C. Persistent AF episodes with average duration of 7 days (10,080±1,440 min). Note the different AF burdens shown for persistent AF, given the nature of the arrhythmia.

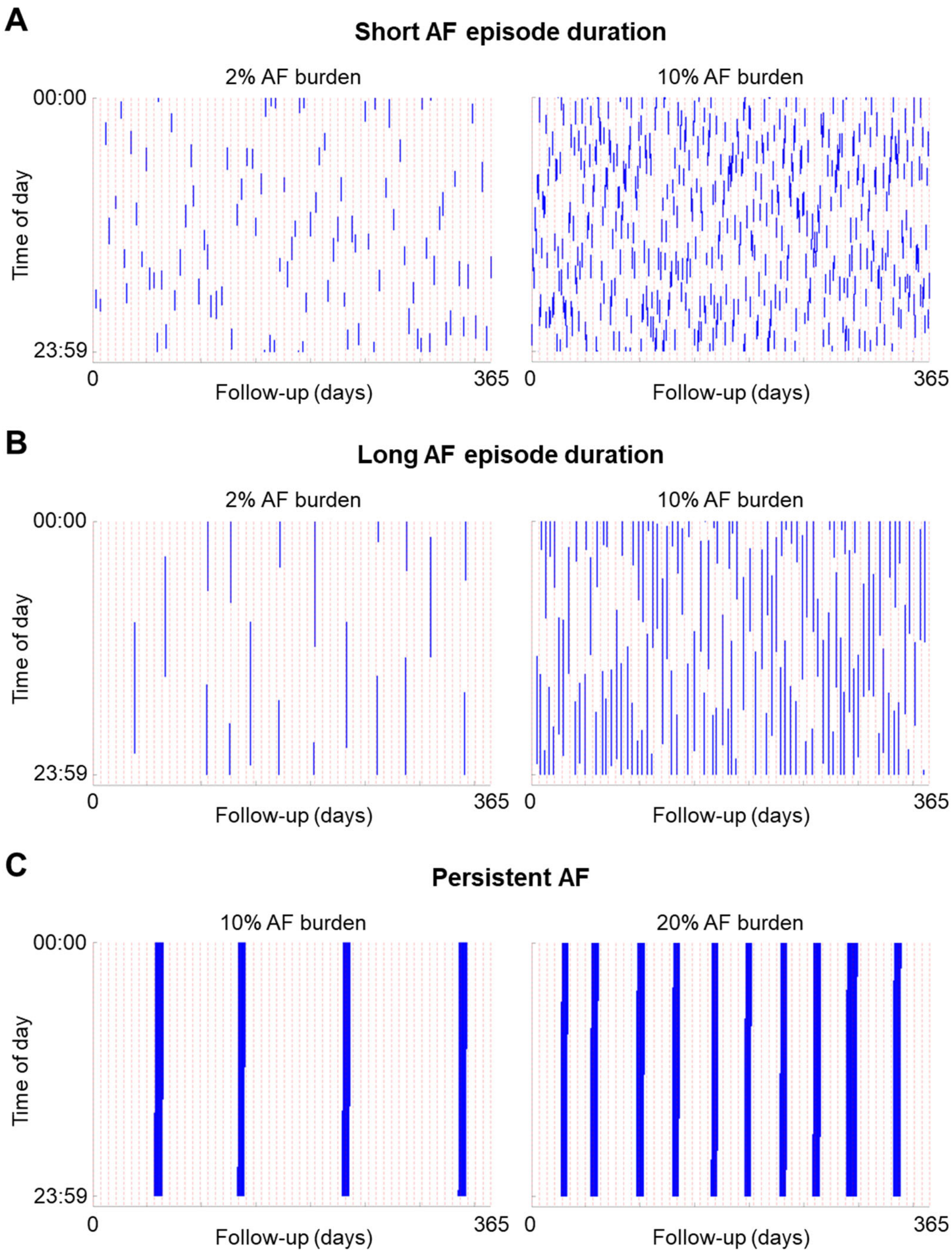

Supplemental Figure 7: AF burden distribution used to determine the accuracy of AF burden estimation based on the percentage of ECGs in AF for the short AF-episode-duration pattern (A), long AF-episode-duration pattern (B), and persistent AF pattern (C). Distribution is based on the estimated distribution from Figure 2.

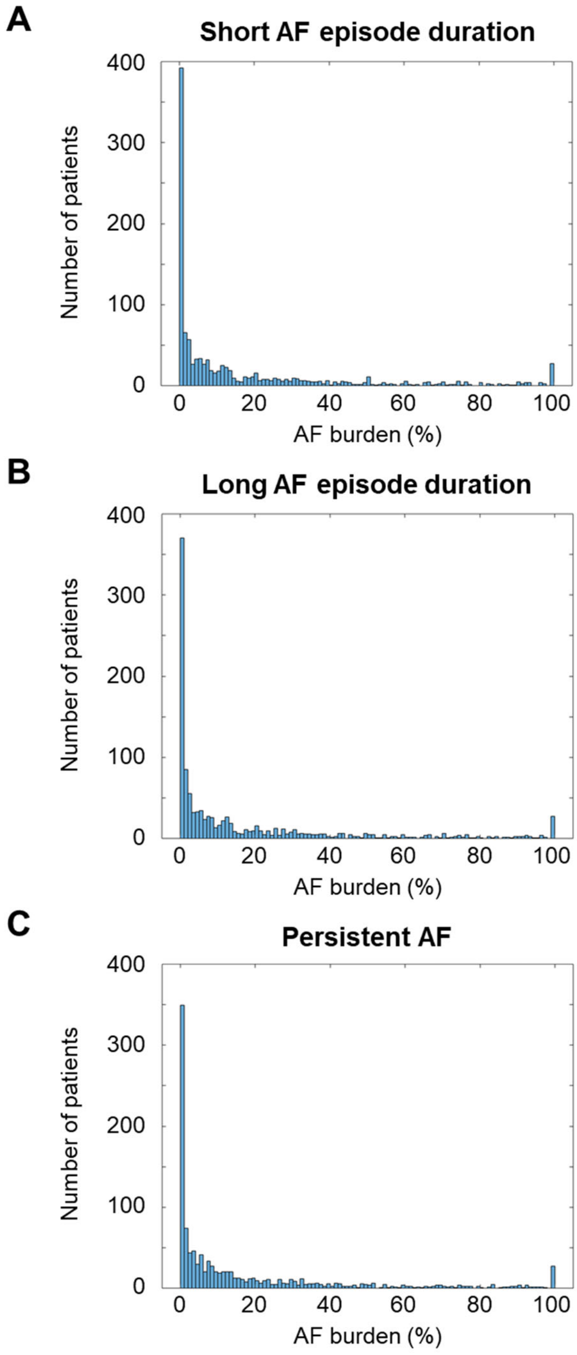

Supplemental Figure 8: Correlation between true AF burden and the percentage of virtual ECGs in AF. Correlation is shown for estimates based on 2 ECGs per week, 1 ECG per week or 1 ECG every 2 weeks (left-to-right) for for the short AF-episode-duration pattern (A), long AF-episode-duration pattern (B), and persistent AF pattern (C).

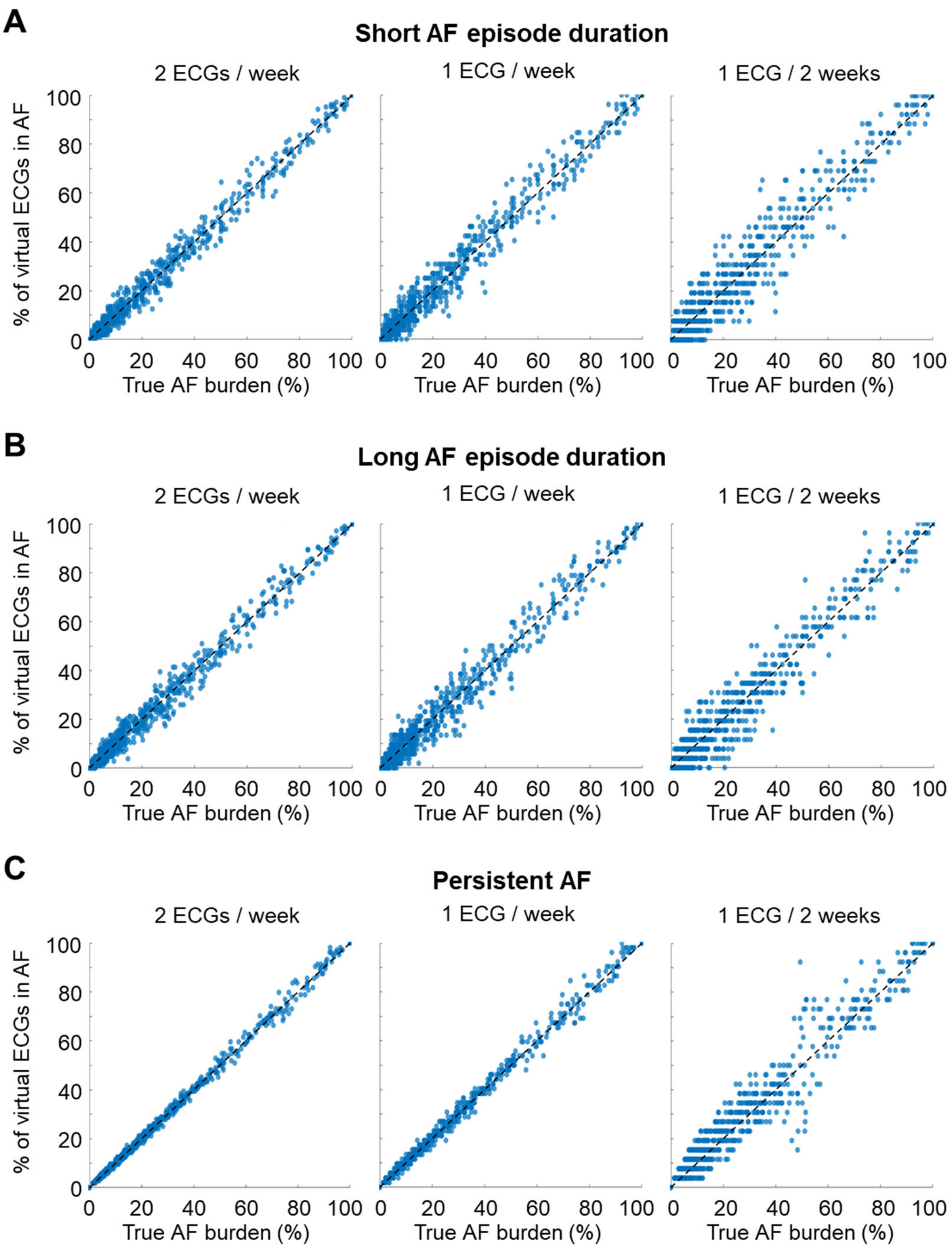

Supplemental Figure 9: Agreement between assigning artificial rhythm patterns to AF burden quartiles based on ECG recording-based estimates and true AF burden. Each square depicts the percentage of rhythm patterns in each of the four AF burden quartiles (Q1: (0,0]%, Q2: (0,5.8]%, Q3: (5.8,21.9]%, or Q4: (21.9,100]%) based on the ECG recording-based AF burden estimate (rows) and true AF burden (columns). Results are shown for estimates based on 2 ECGs per week, 1 ECG per week or 1 ECG every 2 weeks (left-to-right) for for the short AF-episode-duration pattern (A), long AF-episode-duration pattern (B), and persistent AF pattern (C). The numbers in green squares reflect the overall percentage of patterns assigned to the correct AF-burden group based on ECG recordings.

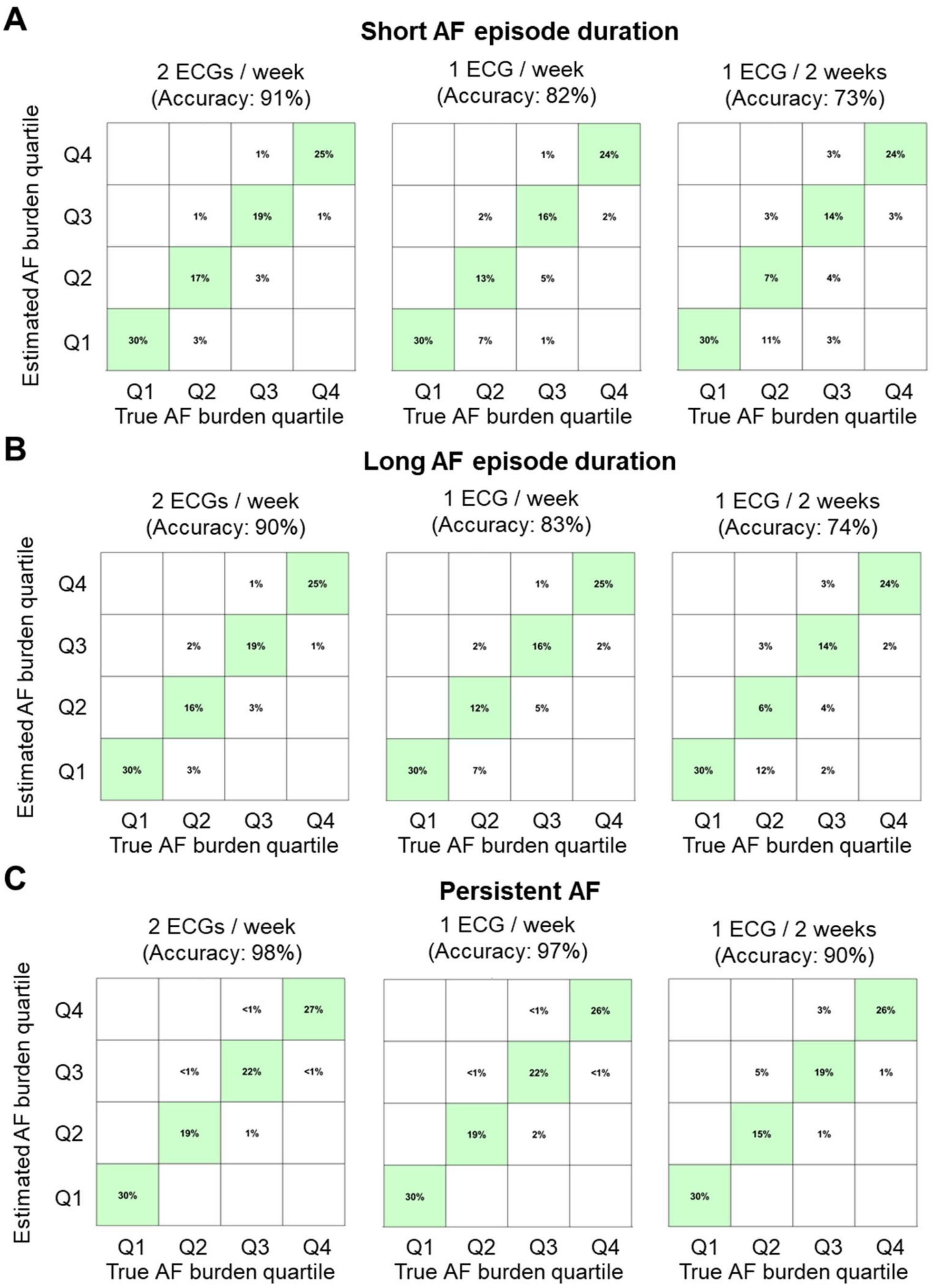

Supplemental Figure 10: Agreement between assigning artificial rhythm patterns to AF burden quartiles based on ECG recording-based estimates and true AF burden. Each square depicts the percentage of rhythm patterns in each of the four AF burden quartiles (Q1: (0,0]%, Q2: (0,5.8]%, Q3: (5.8,21.9]%, or Q4: (21.9,100]%) based on the ECG recording-based AF burden estimate (rows) and true AF burden (columns) in a virtual population comprising 1/3 short AF episodes, 1/3 long AF episodes and 1/3 persistent AF for estimates based on 2 ECGs per week, 1 ECG per week or 1 ECG every 2 weeks (left-to-right). The numbers in green squares reflect the overall accuracy (percentage of patterns assigned to the correct AF-burden group based on ECG recordings).

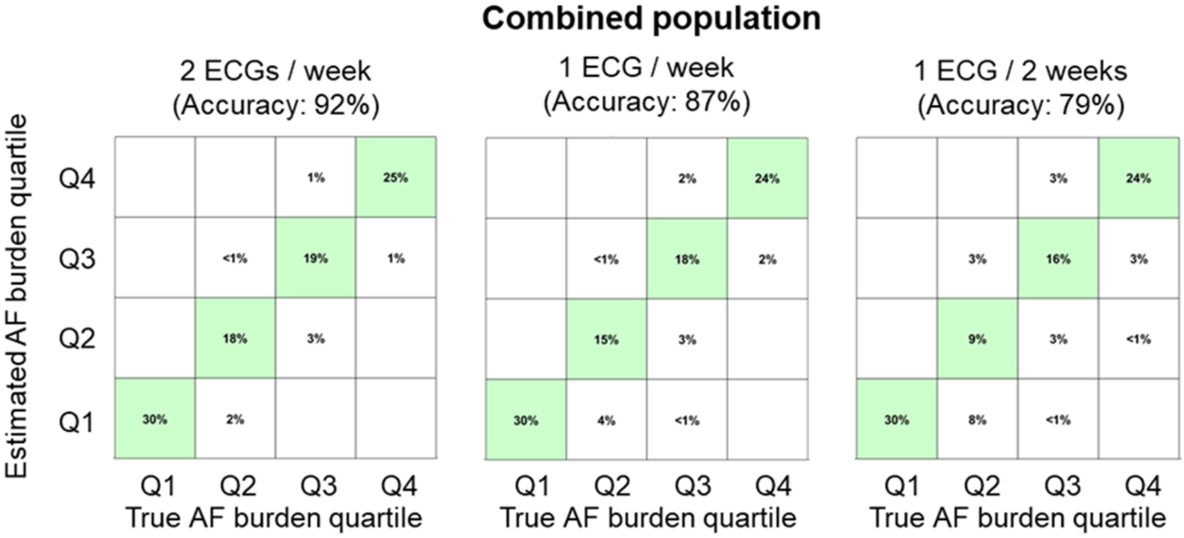

Data availability

Data will be made available upon request. Please contact info@kompetenznetz-vorhofflimmern.de.

Further funding

U.S. received research grants to institution from EU, Roche, and the Dutch Heart Foundation.

J.O. received research grants from German Heart Foundation, University of Hamburg, and German Federal Ministry of Education and Research. In addition, J.O. acts as Managing Director of the IDM gGmbH, subsidiary of the University Medical Center Hamburg-Eppendorf.

L.E. received research support from German Heart Foundation.

L.F. received institutional research grants by EU 633196 (CATCH-ME), EU 847770 (AFFECT-EU) and EU 965286 (MAESTRIA). British Heart Foundation (AA/18/2/34218), German Center for Cardiovascular Research supported by the German Ministry of Education and Research (DZHK). In addition, L.F. received institutional research grants from NIHR, Medical Research Council (UK) and DZHK.

M.D.L. received a travel grant from biosense webster and a research grant from Farapulse. In addition, he was supported by the Research Promotion Fund of the Faculty of Medicine (Hamburg, UKE, “Clinician Scientist Program”).

C.M. received research funding from German Center for Cardiovascular Research (DZHK; Promotion of women scientists programme; FKZ 81X3710112), the Deutsche Stiftung für Herzforschung, the Dr. Rolf M. Schwiete Stiftung, NDD, and Loewenstein Medical unrelated to the current work.

A.R. received a research grant from Medtronic.

R.B.S. has received funding from the European Research Council (ERC) under the European Union’s Horizon 2020 research and innovation programme under the grant agreement No 648131, from the European Union’s Horizon 2020 research and innovation programme under the grant agreement No 847770 (AFFECT-EU) and German Center for Cardiovascular Research (DZHK e.V.) (81Z1710103 and 81Z0710114); German Ministry of Research and Education (BMBF 01ZX1408A) and ERACoSysMed3 (031L0239). Wolfgang Seefried project funding German Heart Foundation. Public funding bodies made payments to the institution for grants.

A.S. received grants to the institution for statistical analysis from EU Horizon 2020, Biotronik and Adrenomed AG and a grant from AFNET for statistical analysis.

S.W. received grants Abbott and Boston Scientific outside the submitted work.

A.Z. received grants to the institution for statistical analysis from EU Horizon 2020, Biotronik and Adrenomed AG.

PK was partially supported by European Union MAESTRIA (grant agreement 965286), British Heart Foundation (AA/18/2/34218), German Center for Cardiovascular Research supported by the German Ministry of Education and Research (DZHK, grant numbers DZHK FKZ 81X2800182, 81Z0710116, and 81Z0710110), German Research Foundation (Ki 509167694),

Dutch Heart Foundation (DHF), the Accelerating Clinical Trials funding stream in Canada, and the Else-Kröner-Fresenius Foundation.

Ethical approval

The protocol of EAST-AFNET 4 was approved by the ethics review boards of all the institutions involved. Written informed consent was provided by all patients who participated in the trial.

Ethikkommission der Ärztekammer Westfalen-Lippe und der Medizinischen Fakultät der Westfälischen Wilhelms-Universität Münster, 09/02/2011, ref: 2010-274-f-A

Pre-registered clinical trial number

ISRCTN: ISRCTN04708680

ClinicalTrials.gov: NCT01288352

EudraCT: 2010-021258-20
